# Cancer Prevention and Treatment with Polyphenols: Type IV Collagenase-Mediated Mechanisms

**DOI:** 10.3390/cancers16183193

**Published:** 2024-09-19

**Authors:** Wojciech Pawłowski, Miłosz Caban, Urszula Lewandowska

**Affiliations:** Department of Biochemistry, Faculty of Medicine, Medical University of Lodz, Mazowiecka 5, 92-215 Lodz, Poland; wojciech.pawlowski1@student.umed.lodz.pl (W.P.); milosz.caban@student.umed.lodz.pl (M.C.)

**Keywords:** polyphenols, anti-cancer, anti-oxidant, chemoprevention, gelatinases, MMP, NF-κB

## Abstract

**Simple Summary:**

Natural polyphenols are well-known dietary supplements that have been used for medical purposes for a long time. Consuming foods and beverages of plant origin, especially those rich in polyphenolic compounds, may have chemopreventive and therapeutic effects on cancer due to the health-promoting properties of these compounds. Polyphenols are characterized by high structural diversity, which contributes to their wide range of therapeutic effects, including anti-oxidant and anti-cancer activities. These compounds can modulate the expression and activity of many proteins, including enzymes, and regulate multiple cell signaling pathways. Among the molecular targets of anti-cancer agents are matrix metalloproteinases, particularly metalloproteinase-2 and metalloproteinase-9, and nuclear factor-kappa B. Matrix metalloproteinases can degrade the protein components of the extracellular matrix and play key roles in physiological processes such as tissue repair and morphogenesis, as well as in carcinogenesis and other pathological processes. The inhibition of their synthesis and activity is closely related to a reduction in the metastasis and invasion of cancer cells. This review discusses the current state of knowledge concerning the anti-invasive and anti-metastatic potential of selected polyphenols, with a focus on in vitro and in vivo evidence.

**Abstract:**

Polyphenols are natural compounds found in many plants and their products. Their high structural diversity bestows upon them a range of anti-inflammatory, anti-oxidant, proapoptotic, anti-angiogenic, and anti-metastatic properties, and a growing body of research indicates that a polyphenol-rich diet can inhibit cancer development in humans. Polyphenolic compounds may modulate the expression, secretion, or activity of compounds that play a significant role in carcinogenesis, including type IV collagenases, such as matrix metalloproteinase-2 (MMP-2) and matrix metalloproteinase-9 (MMP-9), by suppressing cellular signaling pathways such as nuclear factor-kappa B. These enzymes are responsible for the degradation of the extracellular matrix, thus promoting the progression of cancer. This review discusses the current state of knowledge concerning the anti-cancer activity of polyphenols, particularly curcumin, resveratrol, epigallocatechin-3-gallate, genistein, and quercetin, with a specific focus on their anti-invasive and anti-metastatic potential, based on the most recent in vitro and in vivo studies. It appears that polyphenols may be valuable options for the chemoprevention and treatment of cancer via the inhibition of MMP-2 and MMP-9 and the suppression of signaling pathways regulating their expression and activity.

## 1. Introduction

Cancer presents significant problems for health care worldwide. The most recent data from GLOBOCAN 2022, prepared by the International Agency for Research on Cancer, indicate that 20 million new cases of cancer occurred globally in 2022, with approximately 10 million cancer-related deaths also being reported [1]. Unfortunately, it is estimated that the incidence of cancer will increase by about 77% by the year 2050 [1]. Unfortunately, many anti-cancer drugs are expensive, and their use is often associated with severe side effects and complications; in addition, resistance to cancer therapy is becoming more prevalent [2,3]. Regrettably, many cancers are diagnosed at an advanced stage of disease, when the efficacy of therapy is limited [4,5]; hence, there is a need for more preventive and therapeutic strategies and to identify new agents that could lower the burden of cancers. 

To effectively restrict the development and progression of cancer, any potential chemopreventive agents should be able to inhibit cell viability and migration and demonstrate anti-invasive and anti-metastatic potential. A key step responsible for the invasion and migration of cancer cells is the remodeling of the extracellular matrix (ECM) [6,7]. This tumorigenic process can be divided into three stages: ECM deposition, modification, and degradation. Firstly, cancer-associated fibroblasts deposit collagen and other components, such as fibronectin, laminin, and hyaluronic acid. Then, biochemical modifications of the existing ECM structure take place, consisting of fibrosis, increased ECM stiffness, and the creation of cell migration channels. Finally, matrix metalloproteinases (MMPs) and other enzymes cause the degradation of the ECM structure, which promotes tumor proliferation, migration, invasion, and angiogenesis [7]. Therefore, MMPs play a key role in this process. In particular, the expression and activity of MMP-2 and MMP-9, also known as gelatinases or type IV collagenases, are correlated with the aggressiveness of cancer and a poor prognosis [8,9]. 

These enzymes degrade ECM components, primarily type IV collagen, enabling cell migration and the formation of pathological blood vessels and metastases [10]. Thus, MMPs are recognized as boosters of tumor growth [11]. Their expression is known to be regulated by certain signaling pathways, mainly nuclear factor-kappa B (NF-κB) or mitogen-activated protein kinases/extracellular-signal-regulated kinases (MAPK/ERK) [12]. Therefore, potential chemopreventive agents should not only reduce the expression and activity of MMP-2 and MMP-9 but also modulate cellular signaling pathway activation.

For centuries, natural compounds of plant origin have been used to treat diseases. Recently, their influence on carcinogenesis has been intensively studied to improve the difficult and challenging process of managing cancer. They are generally characterized by minor side effects and high safety but also, unfortunately, low water solubility and bioavailability. One of the methods to overcome these disadvantages is the use of modern nanodrug delivery systems [7]. Plant-derived compounds, specifically polyphenols—the subject of this review—are suggested to play an important role in preventing cancer progression as MMP, especially MMP-2 and MMP-9, inhibitors [13]. 

Natural compounds have also been shown to regulate the epithelial–mesenchymal transition (EMT). This term refers to morphological changes in epithelial cells that gain phenotypic characteristics of mesenchymal cells. The EMT includes the deconstruction of epithelial cell-to-cell bonds, the loss of apical–basal polarity, the rebuilding of the actin cytoskeleton, the general prevalence of mesenchymal over epithelial markers, and the increased expression of MMPs. All of these changes occur at the very beginning of cancer development and are responsible for enhanced cell motility, the acquisition of the ability to invade and consequently form distant metastases, and even increased therapeutic resistance [14,15,16]. Therefore, an attempt to reverse EMT changes, especially those associated with MMPs, could be an attractive way to treat cancer. While studies on synthetic MMP inhibitors, such as marimastat, rebimastat, and tanomastat, failed due to their strong adverse effects, phytochemicals, devoid of such properties, offer an alternative to them [11,15]. 

**Figure 1:**
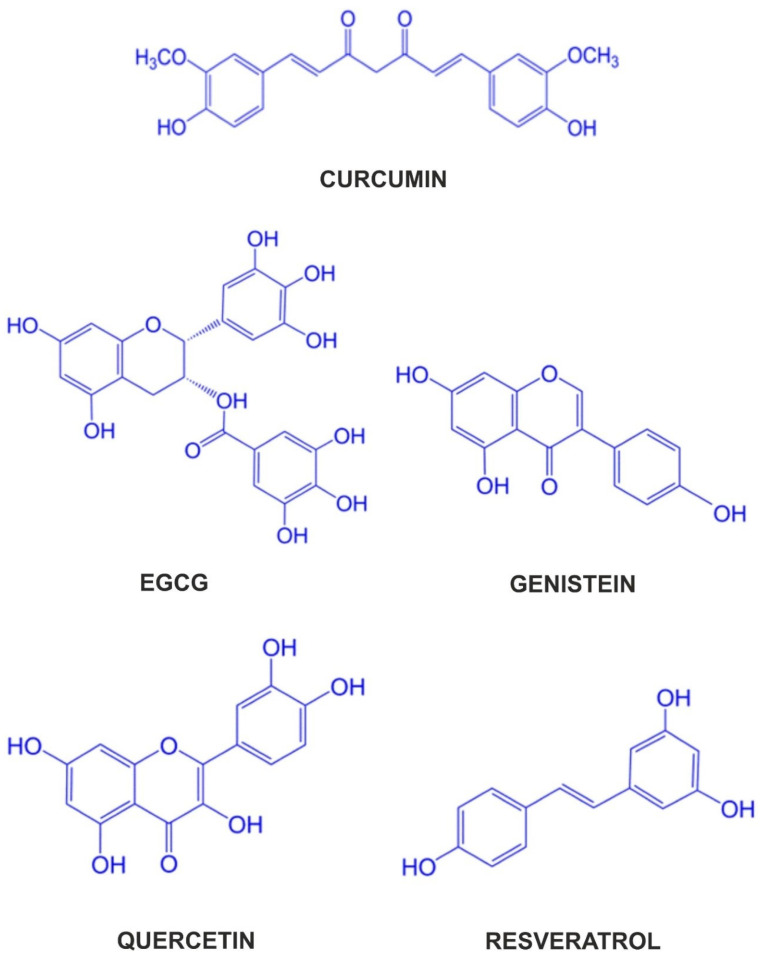
Chemical structures of polyphenolic compounds. Curcumin (1,7-bis(4-hydroxy-3-methoxyphenyl)-1,6-heptadiene-3,5-dione); genistein (4′,5,7-trihydroxyisoflavone); EGCG (2 R,3 R)-3′,4′,5,5′,7-pentahydroxyflavan-3-yl 3,4,5-trihydroxybenzoate); quercetin (3,3′,4′,5,7-pentahydroxyflavone); resveratrol (3,5,4′-trihydroxystilbene).

This review provides a comprehensive overview of the anti-cancer potential of the most common single polyphenolic compounds, such as curcumin, resveratrol, epigallocatechin-3-gallate (EGCG), quercetin, and genistein (Figure 1), known to modulate the activity and expression of MMP-2 and MMP-9. We selected these polyphenolic compounds due to their various unique pro-health properties and their diverse sources of origin. A brief description of these compounds is provided in Table 1. It also examines the current state of knowledge regarding cellular signaling pathways associated with their expression and activity, particularly the NF-κB pathway, which play roles in the metastasis and invasion of cancers. 

Numerous review articles describing the potential utility of polyphenols for cancer prevention and therapy have been published [7,10,14,15,16,17]. Despite this, there is still a need to identify new compounds that could contribute to the prevention of cancer development as well as enrich current forms of cancer therapies. Polyphenols, as compounds of natural origin, seem to be good candidates and are actually the subject of numerous studies. To confirm their efficacy in chemoprevention and cancer treatment, the constant updating of knowledge is necessary. This is the main reason we created this paper. We focus on five main polyphenols, which we examine after analyzing, in detail, the most recent studies on this topic. Also, we provide the mechanisms of their action.

## 2. Search Strategy

The PubMed, Google Scholar, Wiley, Springer, Scopus, Embase, and Web of Science databases were systematically and extensively searched to obtain the collected bibliography. The search included all studies published until May 2024 and used the following keywords, alone or in combination: cancer, cancer diseases, curcumin, EGCG, genistein, quercetin, resveratrol, polyphenols, polyphenolic compounds, chemoprevention, MMP-2, MMP-9, gelatinases, type IV collagenases. Clinical trials were searched for using the ClinicalTrials.gov database. The searches were filtered to include only those studies published in English.

## 3. Polyphenols as Modulators of the Activity and Expression of MMP-2 and MMP-9

Polyphenols are plant secondary metabolites that exhibit strong anti-oxidant properties, thus protecting plants from external stress and danger, for example, insects, as well as UV radiation and drying [18,19]. They are characterized by common structural characteristics, which include at least two phenyl rings and one or more hydroxyl substituents [20]. Polyphenols can be divided into two main groups: flavonoids and nonflavonoids. Flavonoids are, in fact, diphenyl propanes (C6-C3-C6), in which phenolic rings are connected by a heterocyclic ring [20]. According to their structural differences, flavonoids can be further divided into six major subclasses: anthocyanidins, flavanols, flavanones, flavones, flavonols, and isoflavonoids [21]. In turn, nonflavonoids, with a basic structure of only one aromatic ring, consist of many varying subclasses, among which the most important are phenolic acids, stilbenes, and lignans [20,21].

Recent studies indicate that polyphenols can affect various biochemical processes and regulate various intracellular pathways responsible for many pathological conditions, such as inflammation, oxidative stress, angiogenesis, and carcinogenesis [17,22,23,24]. Polyphenolic compounds may downregulate the expression and activity of MMP-2 and MMP-9 by modulating a number of intracellular signaling pathways, such as the NF-κB, phosphoinositide 3-kinase/protein kinase B (PI3 K/Akt), ERK, and MAPK pathways, among others [10,13,25,26]. Some of the most recent in vitro and in vivo studies investigating the effects of natural and synthetic polyphenols on the expression and activity of MMP-2 and MMP-9 in various cancer models are summarized below.

### 3.1. Curcumin

Curcumin (1,7-bis(4-hydroxy-3-methoxyphenyl)-1,6-heptadiene-3,5-dione) is the main natural polyphenol present in Curcuma longa (turmeric) and is widely used as a traditional medical herb in Asian cuisine [27]. It is able to modulate many carcinogenesis-related mechanisms and pathways. For instance, type IV collagenase activity has been found to be dysregulated after curcumin treatment [28]. 

#### 3.1.1. In Vitro Studies

One study evaluated the effect of curcumin treatment on MMP-2 and MMP-9 gene expression in a breast cancer cell line (MDA-MB-231). While low concentrations of curcumin (10 μM) had no effect on MMP gene expression, higher concentrations (20 μM and 40 μM) yielded a significant reduction in MMP expression after 48 and 72 h incubation. This was accompanied by the elevated expression of tissue inhibitors of metalloproteinases (TIMPs), mainly TIMP-1 [29]. TIMPs, in addition to inhibiting MMPs, are also assumed to participate in processes involved in cancer progression and metastasis; in particular, they have been found to inhibit angiogenesis by selectively blocking the action of various growth factors, such as fibroblast growth factor 2 and vascular endothelial growth factor A (VEGF-A) [30]. Lin and co-workers [31] demonstrated that curcumin attenuated the migration and invasion of mouse–rat hybrid retinoblastoma ganglion cells (N18); the authors attribute this to the inhibition of MMP-2 and MMP-9 gene expression and the suppression of various signaling pathways, such as MAPK/ERK and NF-κB. These effects were time- and concentration-dependent. An in vitro study of the anti-tumor activity of curcumin in endometrial adenocarcinoma found that curcumin suppressed cancer cell migration, accompanied by a significant reduction in MMP-2 and MMP-9 protein expression [32]. 

In a study by Zhao and co-workers [33] on the hepatocellular carcinoma cell lines HepG2 and SK-Hep-1, Western blot analysis showed a significant reduction in MMP-2 and MMP-9 expression, resulting in strong anti-proliferative, anti-metastatic, and anti-invasive effects. Jia et al. [34] demonstrated the anti-cancer properties of curcumin in Wilms’ tumor (WT) cells extracted from patients, this being the most common kidney tumor in children. When administered at a concentration of 20 mM, curcumin caused a significant reduction in MMP-2 and MMP-9 at the mRNA and protein levels. It was associated with decreased reversion-inducing cysteine-rich protein with Kazal motifs methylation and reduced proliferative, invasive, and migratory capabilities of the cells in vitro. Another study found curcumin to enhance the anti-cancer activity of metformin, with combined administration yielding stronger effects. However, the curcumin-related reduction in MMP-2 and MMP-9 protein levels was minimal in vitro, which might have resulted from the fact that very small concentrations were used (2.5 or 5 μM). Treatment also downregulated the PI3 K/Akt, NF-κB, and mammalian target of rapamycin (mTOR) signaling pathways, as well as the migration and invasion of hepatocellular carcinoma (HCC) cells [35].

Selected studies investigating the beneficial effects of curcumin in vitro are summarized in Table 2. 

#### 3.1.2. In Vivo Studies 

Numerous in vivo studies have demonstrated the ability of curcumin to suppress MMPs and subsequently limit the aggressiveness of tumors. Zhu et al. [37] examined the effect of curcumin on acute monocytic leukemia (AML) SHI-1 cells using a subcutaneous xenograft model in female severe combined immunodeficient (SCID) mice. Fifteen-day intraperitoneal therapy with curcumin (15 and 30 mg/kg) in a stand-alone treatment contributed to a significant decrease in tumor volume and weight. In addition, hematoxylin and eosin staining showed signs of intense tumor cell damage. Moreover, curcumin inhibited cell proliferation and enhanced cancer cell apoptosis within the tumor. Most significantly, curcumin suppressed the expression of MMP-2 and MMP-9 at the mRNA and protein levels in a dose-dependent manner, preventing AML metastasis. This decrease in MMP levels was confirmed by immunohistochemical staining. These changes were associated with the downregulation of the NF-κB pathway and the modulation of the MAPK pathway. In endometrial adenocarcinoma, immunohistochemical analysis of xenograft tumor sections in female six-week-old non-obese diabetic SCID (NOD-SCID) mice previously injected with Ishikawa cells demonstrated that curcumin treatment inhibited the expression of MMP-2 and MMP-9 and limited tumor growth observed after 31 days of treatment, which was not associated with any toxic effects, and the mean body weight of the animals remained stable [32].

In a study by Zhao and co-workers [33], hepatoma cells were injected into female Balb/c-nu nude mice to induce subcutaneous tumors, and HepG2 cells were intravenously injected into the caudal vein to create a metastatic tumor model. Curcumin treatment led to a dose-dependent reduction in tumor growth and final volume and a reduction in the number of tumor nodules in the lungs. Reduced MMP-2 and MMP-9 expression was noted in the tumor tissue, inhibiting the invasion and metastasis of neoplastic cells. Wang and co-workers [36] conducted an interesting study evaluating the effect of curcumin on the psychological stress-induced proliferation and invasion of glioma cells. LN229 cells were implanted into female Balb/c nu/nu mice. An adverse stress model was created by placing the animals in a 50 mL multi-well centrifuge without squeezing their bodies. It was found that the MMP-2 and MMP-9 levels in tumor tissue, epinephrine and norepinephrine levels in serum, and the tumor volume were significantly higher in the stressed group than in the non-stressed group. The curcumin treatment reversed these stress-related negative effects and reduced the protein expression of MMPs by half in the non-stressed control group. Due to the ability of curcumin to cross the blood–brain barrier, it may be a promising agent for alleviating the progression of glioma due to psychological stress. Also, in the case of WTs, the mean volume and weight of the tumors in vivo were noticeably lower after 21 days of curcumin treatment [34]. In a model of hepatocellular carcinoma in female Balb/c nu mice, 60 mg/kg curcumin treatment decreased MMP expression by approximately 25% in the xenografts, with the effect being enhanced by the addition of metformin [35]. 

As curcumin is characterized by low water solubility (hydrophobic), an unstable chemical structure, poor absorption, and rapid metabolism, the oral administration of its pure form does not typically yield significant health-promoting results. Hence, in recent years, studies have evaluated the use of curcumin administered in the form of nanoparticles, liposomes, and micelles. Such methods of delivering the polyphenol directly to target tissues in a living organism have yielded very promising results [38]. Tavakoli and co-workers [39] examined the effects of 12-day administration of pure (15 mg/kg) and nano-encapsulated (30 mg/kg) curcumin in a mouse B16 F10 melanoma tumor model using C57 B16 mice. In both animal groups, the expression of MMP-2 and MMP-9 genes was significantly decreased in tumor tissues, while the TIMP-1 and TIMP-2 genes were overexpressed. Although both forms inhibited tumor growth, the nano-encapsulated curcumin showed a stronger effect. These data confirm that curcumin may impair tumor growth and reduce the metastatic ability of melanoma in vivo by inhibiting MMPs and by stimulating their inhibitors.

Similar results were obtained in another in vivo study by Sesarman et al. [40], who examined the effects of curcumin, alone or combined with doxorubicin, on C26 murine colon carcinoma using male Balb/c mice with subcutaneously injected cancer cells. The animals intravenously received 5 mg/kg curcumin and 2.5 mg/kg doxorubicin in long-circulating liposomes or in free form on days 7 and 10 after tumor cell inoculation. Curcumin in its pure form led to an almost identical reduction in tumor size to that obtained with doxorubicin, i.e., a decrease of approximately 30% compared to controls. Both compounds yielded more significant results in liposomal encapsulation, i.e., about a 70% reduction in the case of curcumin. However, the highest anti-tumor efficacy was achieved when both of them were administered simultaneously, i.e., about 85%. This confirms that curcumin is able to enhance the anti-cancer properties of chemotherapeutics in vivo. The research group identified potential mechanisms. Free curcumin had no significant effect on the activity of either MMP-2 or MMP-9, although MMP-2 activity slightly increased. In turn, liposomal curcumin reduced the activity of MMP-9 by more than half, without any effect on MMP-2. Moreover, curcumin presented an anti-oxidant effect and inhibited the NF-κB pathway and AP-1, reducing the ability of the tumor to metastasize [40]. 

Selected studies investigating the beneficial effects of curcumin in vivo are summarized in Table 3.

### 3.2. Epigallocatechin-3-Gallate

Epigallocatechin-3-gallate (2 R,3 R)-3′,4′,5,5′,7-pentahydroxyflavan-3-yl 3,4,5-trihydroxybenzoate) is an important polyphenol occurring in green tea, prepared by brewing leaves from Camellia sinensis. EGCG affects a wide range of processes in humans. It is believed that EGCG may exert anti-oxidative, anti-cancer, and anti-inflammatory activities, as well as decrease blood lipids and glucose, increase insulin sensitivity, or reduce body weight [41,42]. It modulates the activity of genes and various signaling pathways involved in cell proliferation, apoptosis, and the inhibition of cancer, such as pro- and anti-apoptotic proteins (B-cell lymphoma 2 (Bcl-2), Bcl-2-like protein 4 (Bax), B-cell lymphoma-extra large (Bcl-XL)), MAPK/ERK, PI3 K/Akt, and NF-κB, and is involved in the epigenetic downregulation of oncogenes [43]. EGCG was found to have the greatest anti-proliferative effect among 10 representative green tea polyphenols [44] and has long been known to inhibit MMP-2 and MMP-9 expression [45,46,47]. 

#### 3.2.1. In Vitro Studies 

Multiple studies have demonstrated that EGCG has an inhibitory effect on type IV collagenase expression in cancer cell lines. Roomi and colleagues [48] assessed the role of EGCG in the regulation of MMP-2 and MMP-9 in human melanoma A-2058 cells using a gelatinase zymography assay and densitometry analysis. The cells were incubated with EGCG alone; EGCG together with phorbol 12-myristate 13-acetate (PMA), a potent tumor promoter; and PMA alone. Melanoma A-2058 is characterized by elevated secretion of both MMP-2 and -9 in comparison to normal cells. Treatment with 100 ng/mL PMA did not affect MMP-2 expression but significantly increased MMP-9 expression. Various concentrations of EGCG (10, 25, 50, 100 μM) inhibited MMP-2 and MMP-9 expression in a concentration-dependent manner, with a 57% reduction noted for MMP-2 and 73% for MMP-9 at the maximum concentration (100 μM).

Similar results were obtained in head and neck squamous carcinoma cells (FaDu) and tongue carcinoma cells (SCC-25). Quantitative densitometry analysis of gelatinase zymography revealed that EGCG (10 μM, 25 μM, 50 μM, 100 μM) decreased both MMP-2 and MMP-9 activity in a concentration-dependent manner [49]. Also, EGCG (10 μM, 25 μM, 50 μM, 100 μM) was found to block the activity of MMP-2 and MMP-9 in pediatric human sarcomata (osteosarcoma U2 OS and rhabdomyosarcoma RD) [50], adult human sarcomata (chondrosarcoma SW-1353, fibrosarcoma HT-1080, liposarcoma SW-872, synovial sarcoma SW-982) [51], the human cervical cancer lines HeLa and DoTc2-4510, the human ovarian cancer line SK-OV-3 [52], and human renal cell carcinoma 786-0 (EGCG in concentrations 10 μM, 50 μM, 100 μM, 200 μM) [53]. Multiple studies indicate that EGCG possesses anti-migratory and anti-cancer properties associated with the downregulation of MMP-2 and MMP-9. In human nasopharyngeal carcinoma cells (NPC-39, HONE-1, NPC-BM), EGCG treatment significantly lowered the expression and activity of MMP-2 and reduced the motility and invasion potential of tumor cells by half. The effect was most evident at a concentration of 50 μM EGCG, at which the activity of MMP-2 was reduced by about 70% in HONE-1 and NPC-BM and 50% in NPC-39. [54]. Likewise, EGCG at concentrations of 20 μM or more was found to inhibit, by about half, the expression and activity of MMP-2 and MMP-9 in human renal carcinoma cells (786-0 and ACHN) and thus reduce their migratory and invasive potential [55].

In cholangiocarcinoma, EGCG treatment decreased the enzymatic activity of MMP-2 and MMP-9 in a concentration-dependent manner, inhibited the invasion and migration capacity, and accelerated the apoptosis of HuCC-T1 cells [56]. In addition, EGCG inhibited the expression of MMP-9 at the mRNA and protein levels and downregulated the NF-κB pathway, as well as suppressed the migration, invasion, and proliferation of SW780 cells and induced their apoptosis. Most importantly, it did not exert any significant cytotoxic activity against normal human bladder epithelial cells [57]. EGCG loaded with poly(lactic-co-glycolic acid) (PLGA) nanoparticles suppressed the NF-κB pathway and inhibited the mRNA expression of several downstream molecules, such as MMP-2, cyclooxygenase-2 (COX-2), and tumor necrosis factor α (TNF-α), in the lung cancer cell lines A549 and H1299. PLGA-encapsulated EGCG was far more effective than EGCG in its free form at the same concentration (12.5 μM, 25 μM) [58]. A previous study found that combined therapy with EGCG and a specific NF-κB inhibitor (BAY11-7082) had greater synergistic anti-tumor effects compared to individual drugs. MMP-2 expression was suppressed at the mRNA level in A549 and H1229 cells [59]. In CL1-5 cells, another lung cancer cell line characterized by high invasiveness, EGCG treatment inhibited MMP-2 and MMP-9 at the mRNA and protein levels and reduced enzymatic activity; it also suppressed MMP-2 promoter activity in a concentration-dependent manner and reduced the nuclear translocation of NF-κB and cell invasion and migration [60]. A study by Fang et al. [61] found that EGCG decreased the activity of MMP-2 and MMP-9 in Epstein–Barr virus-negative and -positive nasopharyngeal carcinoma (NPC) cell lines in a concentration-dependent manner, probably by modulating the ERK and NF-κB pathways. MMP-2 expression was also diminished at the mRNA level. As a result, the migration and invasion capabilities of cancer cells were diminished. Finally, EGCG has demonstrated anti-cancer activity in oral squamous cell carcinoma. EGCG decreased the activity of MMP-2 and MMP-9 and the mRNA and protein expression of MMP-2. In addition, TIMP-2 activity and expression were elevated, and the NF-κB and urokinase plasminogen activator (uPA) pathways, known to upregulate MMPs, were suppressed. The cells were found to have reduced motility and invasive and migratory potential, as indicated by the transwell cell invasion and wound-healing migration assays. The authors also noted a decrease in EMT markers [62]. All of these results confirm EGCG’s ability to suppress cancer formation, mainly by modulating MMP-2, MMP-9, and associated pathways. 

Selected studies investigating the beneficial effects of EGCG in vitro are summarized in Table 4. 

#### 3.2.2. In Vivo Studies 

The anti-cancer properties of EGCG have been demonstrated in vivo. Shankar and co-workers [63] proved that EGCG was able to inhibit the growth of human pancreatic tumors in an animal model. Briefly, human PANC-1 cells were injected into the pancreas of Balb/c nu/nu mice, and EGCG administration (60, 80, and 100 mg/kg body weight) was begun one week after implantation. The polyphenol inhibited tumor growth in a dose-dependent manner. Interestingly, no visible signs of toxicity were observed, and the mean weight of the mice increased during the treatment. The treatment decreased MMP-2 and MMP-7 mRNA expression, reduced VEGF protein expression, and downregulated the ERK and PI3 K/Akt pathways in the tumor tissue, attenuating the metastatic ability of the cancer cells. 

In the in vivo model of cholangiocarcinoma, the tumor xenograft model was established by the subcutaneous implantation of HuCC-T1 cells into the backs of Balb/c male mice. The polyphenol was subcutaneously administered beside the solid tumor at a dose of 20 mg/kg of body weight. The treatment effectively inhibited the expression of MMP-2 and MMP-9 in tumor tissue, assessed by immunohistochemical staining, and significantly decreased the tumor volume, indicating that EGCG has strong anti-cancer activity in vivo [56]. 

In another study, the polyphenol was used to treat female Balb/c mice injected with SW780 bladder cancer cells. EGCG was intraperitoneally administered at different doses (25, 50, and 100 mg/kg of body weight). The tumor volume and weight decreased dose-dependently. EGCG also inhibited the expression of MMP-9 at the mRNA and protein levels and downregulated the NF-κB pathway in the tumor lysate [57]. Zhang et al. [58] found EGCG loaded with PLGA to possess beneficial properties against human lung cancer in a patient-derived tumor xenograft model. Firstly, small (2–3 mm^3^) portions of the patient’s resected tumor were injected into male NOD-SCID mice aged eight to ten weeks. Then, when the tumors reached a size of about 1000 mm^3^, they were implanted into other Balb/c athymic mice, which were intraperitoneally treated with 10 mg/kg EGCG (free form) or 5 mg/kg (PLGA-encapsulated). The treatment resulted in a reduced tumor volume and weight and lower phospho-NF-κB expression, as indicated by immunoblot analysis. Importantly, encapsulated EGCG clearly suppressed tumor growth more than free EGCG, proving its greater effectiveness in vivo. Interestingly, a combination of EGCG and BAY11-7082 inhibited the growth of lung cancer tumors in vivo more potently than EGCG alone. Hence, supplementing the polyphenol with a specific inhibitor of the NF-κB pathway appears to have stronger effects and can hence reduce potential side effects by allowing lower doses to be used [59]. 

Moreover, after the injection of CL1-5 cells into Balb/c mice aged six to eight weeks, EGCG treatment reduced cancer invasion and limited tumor metastases in the lungs [60]. In a study by Fang et al. [61], NPC cells were implanted into six-week-old SCID mice that were receiving EGCG at different doses by oral gavage. After eight weeks, all groups treated with the polyphenol developed smaller tumors compared to the control group. The results indicated that EGCG is able to inhibit NPC growth without significant adverse effects. Finally, after subcutaneous implantation of SCC-9 cells into Balb/c nu/nu mice and treatment with 10 mg/kg or 20 mg/kg, EGCG was found to lower tumor weight and volume, indicating its anti-cancer properties in vivo [62]. 

Selected studies investigating the beneficial effects of EGCG in vivo are summarized in Table 5. 

### 3.3. Genistein 

Genistein (4′,5,7-trihydroxyisoflavone) is one of the most commonly known isoflavones. It occurs mainly in soy and soy-based products, which are important elements of Asian cuisine. Its health-promoting properties have been known for a long time, including its anti-cancer, anti-oxidant, anti-inflammatory, anti-angiogenic, and proapoptotic activities [64]. Its activity is believed to be pleiotropic. Genistein has an affinity for estrogen receptors (ERα and ERβ), modifies the expression of various genes and proteins, and modulates various intracellular signaling pathways, including protein tyrosine kinases, NF-κB, PI3 K/Akt, and those associated with apoptosis. However, these beneficial properties depend on the concentration, tissue type, and quantity of ERα. High concentrations of genistein may elicit negative effects in vivo, such as intensified cell proliferation [65,66].

#### 3.3.1. In Vitro Studies 

The anti-cancer and anti-metastatic properties of genistein result largely from its ability to inhibit MMP-2 and MMP-9, as proven in many studies on various cancer cell lines. Shafiee et al. [67] examined the effect of genistein on PC3 prostate cancer cells; genistein treatment caused a significant reduction in MMP-2 activity, as measured by gelatinase zymography. At a concentration of 70 μM, MMP-2 activity was reduced by approximately 70%, and the gene expression of p38 MAPK and its active phosphorylated form were downregulated. In addition, the activity of proapoptotic protein caspase-3 was increased. As a consequence, cell viability and metastatic capability were both reduced. Similar effects were observed for colon cancer cells (HT-29). Although the cells demonstrated a slightly more dynamic decrease in MMP-2 activity after genistein treatment than PC3, the two cell lines presented comparable maximal effects at a concentration of 70 μM [68].

Genistein can inhibit the expression of MMP-2 and MMP-9 at both the mRNA and protein levels and can increase the expression of TIMP-1 in HT29 cells. Treatment with 20 μM genistein achieved a 50% decrease in lytic activity by MMP-2 and MMP-9. Also, genistein treatment downregulated the Wnt-1/β-catenin pathway and upregulated E-cadherin expression, exerting strong anti-migratory and anti-invasive effects against colorectal cancer cells [69]. Likewise, genistein treatment inhibited MMP-9 expression and increased the expression of TIMP-1 in HeLa cervical carcinoma cells [70]. In addition, 5 μM genistein inhibited MMP-2 and MMP-9 expression in Mia-PaCa2 pancreatic cancer cells, as assessed by Western blotting, in a time-dependent manner. This effect resulted in a significant deterioration in cell migration [71].

Genistein also induced a concentration-dependent reduction in the secretion and expression of MMP-2 and MMP-9 in melanoma 518 A2 cells, with a stronger effect than two other flavones: chrysin and apigenin. It has also been reported that combining a polyphenol with a metal complex can intensify its biological effect [72]. Indeed, it has been proven that Cu(II)–genistein inhibited the expression and secretion of MMP-2 and MMP-9 several times more effectively, with greater anti-metastatic potential than genistein alone. These results indicate that compounds of polyphenols with metals may mitigate the development of cancer cells more effectively than their native forms [73]. Some studies reported that genistein suppresses the activity of MMP-2, but not MMP-9. Despite this, genistein treatment still reduced the proliferation of A549 non-small-cell lung carcinoma (NSCLC) cells [74] and impaired invasion, metastasis, and the progression of the EMT induced by transforming growth factor β1 (TGF-β1) in Panc-1 human pancreatic cancer cells [75]. Xiao and co-workers [76] found genistein to inhibit mRNA and protein expression of MMP-2 in colorectal cancer cells. Treatment also significantly reduced the migratory and invasive potential of the cells and their viability. Also, in liver cancer cells, genistein diminished cisplatin-induced stimulation of MMP-2 and slowed down cell proliferation [77]. 

Selected studies investigating the beneficial effects of genistein in vitro are summarized in Table 6. 

#### 3.3.2. In Vivo Studies 

Kidani and co-workers [78] assessed the anti-cancer potential of genistein against osteosarcoma. Osteosarcoma LM8 cells were subcutaneously injected into male Balb/cA Jcl-nu nude mice and male C3 H mice. Before implantation, the study group cells had been incubated for three days with 50 μM genistein, whereas the controls had not. The animals were fed with laboratory chow and water and did not receive genistein during the experiment. The results showed that cells pretreated with the polyphenol presented a different phenotype. In the genistein group, the mice generally developed about four-times smaller tumors than controls, with one mouse not developing a tumor at all. Likewise, in the genistein group, the majority of animals did not demonstrate metastatic nodules in the liver and lungs. Immunohistochemical staining revealed significantly lower levels of MMP-2 in primary tumors in the genistein group, indicating that genistein attenuated the metastatic potential of osteosarcoma. 

Colorectal cancer cells HCT116-LUC were subcutaneously injected into athymic Balb/c mice aged six to eight weeks. When the tumors developed, they were isolated, cut into small pieces, and transplanted into the cecal wall. This applied model of orthotopic implantation intimately reflects the course of cancer progression in humans, as neoplastic cells have to overcome the intestinal wall, initiate angiogenesis, and form distant metastases. Genistein was orally administered using doses of 25 and 75 mg/kg daily. Significantly fewer metastatic foci were observed in the lungs and livers of the animals treated with genistein compared to the control group. An immunohistochemical assay showed a dose-dependent reduction in MMP-2 expression in tumor sections. Genistein also significantly inhibited angiogenesis in vivo [76].

Chen et al. [77] conducted an interesting study in which they evaluated the effect of genistein on liver cancer recurrence after curative hepatectomy. Hepatocellular cancer cells (HCCLM3) were subcutaneously injected into a single nude Balb/c nu/nu mouse. Subsequently, the developed tumor was removed, divided, and implanted into single lobes of the livers of other mice. After 10 days, the tumor-bearing liver lobes were resected. After hepatectomy, all mice in the control group presented intrahepatic recurrence and lung metastasis. In contrast, the genistein treatment halved the tumor volume and decreased the number of metastases in the lungs. In addition, the treatment also enhanced the inhibitory effect of cisplatin on tumor recurrence and eliminated the cisplatin-induced upregulation of MMP-2. Genistein did not have any clear effect on MMP-2 when applied alone, probably due to the rather low dose used, but halved its mRNA expression in tumor sections when administered together with cisplatin. The treatment also inhibited MMP-2 protein expression, measured by immunochemistry.

Selected studies investigating the beneficial effects of genistein in vivo are summarized in Table 7. 

### 3.4. Quercetin 

Quercetin (3,3′,4′,5,7-pentahydroxyflavone) is a flavonol occurring in many fruits, vegetables, leaves, and red wine. It is most commonly found in the form of quercetin glycoside, which is better absorbed by enterocytes. Due to its extremely diverse properties, quercetin has been the subject of extensive research [79]. Quercetin is believed to exert anti-proliferative, anti-inflammatory, anti-carcinogenic, anti-oxidant, anti-bacterial, anti-viral, gastroprotective, radical-scavenging, immune-modulatory, and anti-diabetic activities [80]. In particular, the anti-cancer properties of quercetin result from its ability to promote apoptosis, prevent cell arrest, and suppress metastasis and angiogenesis by modulating various intracellular signaling pathways, such as Wnt/β-catenin, PI3 K/Akt, Janus kinases/signal transducer and activator of transcription proteins (JAK/STAT), MAPK, p53, and NF-κB [81,82].

#### 3.4.1. In Vitro Studies 

A number of in vitro studies have found quercetin to suppress MMPs, which act as key pro-angiogenic and pro-metastasis mediators. In lung cancer, which is responsible for the most cancer-related deaths worldwide, quercetin significantly reduced the protein expression of MMP-2 and MMP-9 and increased TIMP-2 expression. The polyphenol was found to deplete the migration and invasive potential of A549 cells by modulating the Akt/MAPK/β-catenin signaling pathway [83]. Quercetin reversed an increase in MMP-9 expression and activity induced by nickel but did not influence MMP-2; treatment also suppressed the NF-κB and Toll-like receptor 4 (TLR4) pathways [84]. Quercetin also yielded promising treatment results in rapidly infiltrating glioblastoma multiforme, another extremely malignant tumor. Liu et al. [85] revealed that quercetin downregulated MMP-2 and MMP-9 protein expression and significantly inhibited migration and invasion in a time- and concentration-dependent manner; however, a noticeable anti-proliferative effect was noted at a concentration of 20 μg/mL. While at a lower concentration of 10 μg/mL, quercetin did not stop cell proliferation, the polyphenol still significantly attenuated cell migration (30%) and invasion and suppressed both MMPs [86].

It is worth emphasizing that quercetin is able to cross the blood–brain barrier [87], making it a potential new agent for the treatment of brain tumors. Quercetin (50 μM) has also been found to reduce the protein levels of MMP-2 and MMP-9 in oral cancers. Treatment lowered cell migration and invasion by regulating the levels of microRNA-16 (miR-16) and homeobox A10, which are involved in tumor progression. Knock-down of miR-16 abrogated the suppressive effect of quercetin on MMP expression in HSC-6 and SCC-9 cells [88].

Interestingly, it has been shown that quercetin can also attenuate the proliferation, invasion, and migration abilities of osteosarcoma by inhibiting parathyroid hormone receptor 1 and by simultaneously downregulating MMP-2 and MMP-9 and upregulating TIMP-1 and TIMP-2 at the mRNA level; this effect is both time- and concentration-dependent (20–100 μM) [89]. 

The anti-tumor effect of quercetin has also been extensively studied in various gastrointestinal cancers. The administration of quercetin at a concentration of 10 μg/mL decreased the protein expression of MMP-2 and MMP-9, thus inhibiting the migration and invasion of esophageal cancer cells Eca109 [90]. In gastric cancer, quercetin treatment (10 μM for 72 h) also downregulated the expression of MMP-2 and MMP-9, decreasing cell migration and invasion and lowering the protein expression of uPA and the uPA receptor (uPAR). The uPA/uPAR system is considered to play an important role in tumor metastasis. Activated uPA catalyzes the conversion of plasminogen to its active form, plasmin, which, in turn, is responsible for degrading ECM components via the activation of MMPs. In this study, uPA knock-down reduced MMP activity, which was further exacerbated by the quercetin treatment. The authors suggest that quercetin may suppress the uPA/uPAR system by modulating NF-κB, protein kinase C δ, ERK1/2, and 5′-adenosine monophosphate-activated protein kinase α [91].

Quercetin treatment (20 μM, 40 μM, 80 μM) was also found to block MMP-2 and MMP-7 expression in pancreatic cancer [92]. It also suppressed MMPs in metastatic ovarian cancer: treatment (50 μM, 75 μM) inhibited the gene expression, protein expression, and proteolytic activity of MMP-2 and MMP-9, thus attenuating the metastatic potential of ovarian cancer cells [93]. 

Notably, quercetin may exert bidirectional effects in ER-positive breast cancer. Xu et al. [94] showed that at a low concentration (5 μM), quercetin intensified cell proliferation, reduced apoptosis, and even reversed the anti-proliferative effect of tamoxifen to a certain extent. In contrast, at a high concentration (100 μM), quercetin strongly inhibited cell proliferation and acted synergistically with tamoxifen. This may result from the quercetin treatment modulating the mRNA expression of the many genes involved in tumor progression, including MMPs. High concentrations of quercetin significantly decreased MMP-2 and MMP-9 expression, while low concentrations increased it, which, in turn, enhanced the malignant potential of cancer cells. These dual effects of quercetin resulted from its phytoestrogenic properties and the fact that the polyphenol is able to regulate the relative ratios of different ER subtypes in ER-positive breast cancers. Interestingly, it has been shown that quercetin can act synergistically with chemotherapeutic drugs, such as everolimus, doxorubicin, and lonidamine, enhancing their effects, and inhibit MMP-2 and MMP-9 [95,96,97].

Tang and co-workers [98] reported that, in breast cancer cells, quercetin significantly inhibited the protein expression of MMP-2 and MMP-9 and increased the TIMP-1 and TIMP-2 levels in a concentration-dependent manner; it also suppressed the activity of MMP-2 and MMP-9 based on gelatin zymography. Furthermore, quercetin decreased the adhesive, invasive, and migratory abilities of breast cancer cells. These findings were confirmed in another study, which also demonstrated reduced levels of MMP-2 and MMP-9 after treatment with quercetin in MDA-MB-231 breast cancer cells [99]. Also, Balakrishnan and co-workers [100] reported the inhibition of MMP-2 and MMP-9 protein expression in MCF-7 and MDA-MB-231 breast carcinoma cells after quercetin treatment. Likewise, the polyphenol downregulated the phosphorylated epidermal growth factor receptor/vascular epithelial growth factor receptor (p-EGFR/VEGFR) pathway and, subsequently, the PI3 K/Akt pathway. The anti-angiogenic properties of quercetin were proven using the tube formation assay. Also, treatment attenuated the invasiveness and migratory potential of the cells and inhibited the EMT. Also, the polyphenol reduced the protein expression of MMP-2 and uPA and limited cell motility via the inhibition of the Snail/Akt pathway in NSCLC cells [101]. In colorectal cancer cells, quercetin lowered MMP-2 and MMP-9 activity, as confirmed by gelatin zymography. Also, MMPs were inhibited at the mRNA level, whereas their inhibitors, TIMP-1 and TIMP-2, were overexpressed, thus yielding an anti-metastatic effect [102]. Likewise, in HCC cells, the protein expression of MMP-2 and MMP-9 was concentration-dependently inhibited by quercetin. The polyphenol suppressed HCC growth by affecting autophagy and the NF-κB pathway [103]. Quercetin was found to exert inhibitory effects against erlotinib-resistant oral squamous cell carcinoma (OSCC). The erlotinib-resistant cell lines ERL-R5 and ERL-R10 were obtained by maintaining HSC-3 human tongue squamous cell carcinoma cells in media with increasing concentrations of erlotinib for six months. At a concentration of 5 μM, treatment caused a reduction in MMP-2 and MMP-9 at the protein level, which was detected in cell lysates from HSC-3, ERL-R5, and ERL-R10 cells. Also, quercetin inhibited cellular invasiveness and migration and induced apoptosis [104]. 

Selected studies investigating the beneficial effects of quercetin in vitro are summarized in Table 8.

#### 3.4.2. In Vivo Studies 

Pradhan et al. [107] assessed the effect of quercetin on melanoma growth in a mouse model. They subcutaneously implanted B16 F10 melanoma cells into the flanks of four- to six-week-old male C57 BL6 mice. Quercetin at a dose of 15 mg/kg of body weight was injected three times a week into the peripheral parts of the tumors for three weeks. The treatment resulted in the decreased expression of MMP-9 in the tumor sections, as indicated by immunofluorescence analysis, and MMP-9 also demonstrated reduced protein expression and proteolytic activity, as confirmed by Western blot and gelatin zymography assays. As a result, 25% decreases in tumor weight and volume were noted. 

In a study by Tang et al. [98], ZR-75-1 and MCF-7 cells were implanted into male Balb/c nude mice, which were then intraperitoneally treated with 20 mg/kg and 40 mg/kg quercetin. The results indicated that quercetin suppressed tumor growth and did not elicit toxic effects in normal tissues. In a similar study, quercetin suppressed tumor growth and inhibited metastasis and glycolysis via the Akt/mTOR pathway [99]. 

Another study on breast cancer was carried out on female Sprague-Dawley rats. Breast cancer was established using 7,12-dimethylbenz(a)anthracene. After the occurrence of tumors, the rats were treated with intratumoral injections of quercetin (25 mg/kg) or gold-nanoparticle-conjugated quercetin (25 mg/kg) for eight days. In the tumor-bearing animals, both the simple polyphenol and encapsulated polyphenol successfully suppressed tumor growth and restored the mammary epithelial tissue architecture. The anti-angiogenic properties of quercetin were proven using a chick embryo angiogenesis assay [100]. Chang et al. [101] examined the effect of quercetin on NSCLC cells in an orthotopic xenograft model. A549 cells were injected into the left lateral thorax of female SCID mice. The animals were intraperitoneally treated with quercetin (500 mg/kg of body weight). The treatment yielded an anti-metastatic effect in vivo, inhibited tumor growth, and prolonged survival time. Also, quercetin has been found to prevent metastasis formation in colorectal cancer in vivo. An experimental lung metastasis model was established by injecting mouse colon carcinoma CT26 cells into the lateral tail veins of five-week-old Balb/c female mice. The animals treated with quercetin developed fewer tumor nodules in the lungs [102]. Similar results indicating the inhibition of cancer metastasis development in the lungs via the suppression of MMPs have also been confirmed in osteosarcoma and melanoma [105,106]. In another study, H22 human hepatocellular carcinoma cells were subcutaneously injected into four-week-old male Balb/c mice to create a xenograft model. Quercetin was administered through gavage at different doses (25, 50, and 100 mg/kg of body weight). The tumor weight and volume were significantly decreased in animals treated with the polyphenol [103].

Finally, quercetin was found to have beneficial properties in an animal model of erlotinib-resistant OSCC. A tumor model was developed by subcutaneously injecting four-week-old male nude mice with ERL-R5 cells, and the mice were intraperitoneally treated with quercetin (2 or 10 mg/kg) for 18 days. The results showed a dose-dependent depletion in tumor weight and volume after the treatment [104]. 

Selected studies investigating the beneficial effects of quercetin in vivo are summarized in Table 9.

### 3.5. Resveratrol 

Resveratrol (3,5,4′-trihydroxystilbene) occurs naturally in many plants, including peanuts, pistachios, and berries; it is also present in grapes and, thus, red wine. Interest continues to grow in resveratrol as its broad biological properties are being discovered; it is currently recognized as having cardioprotective, anti-oxidative, anti-inflammatory, neuroprotective, anti-diabetic, and anti-tumor properties [108,109]. Various studies have shown that resveratrol affects many stages of carcinogenesis. It plays a role in initiation by reactive oxygen species scavenging, protecting DNA from damage and anti-mutagenic activity; it influences promotion through its proapoptotic and anti-inflammatory properties and by inhibiting cell proliferation via interference with the PI3 K/Akt, mTOR, MAPK, Wnt/β-catenin, and NF-κB signaling pathways; and it also inhibits progression and metastasis via the suppression of MMPs and its anti-angiogenic and anti-migratory properties [110,111]. Hence, resveratrol appears to be a very promising agent in the inhibition of cancer development. In particular, many scientific works indicate that resveratrol slows carcinogenesis by inhibiting MMP-2 and MMP-9.

#### 3.5.1. In Vitro Studies 

In glioblastoma, the most common and lethal malignant tumor of the central nervous system, resveratrol treatment (40 μM) suppressed the protein expression and enzymatic activity of MMP-2 and significantly inhibited the migration and invasion of cancer cells in vitro [112]. Resveratrol has been found to suppress MMPs in various OSCC cell lines, most likely through the inhibition of the MAPK/ERK pathway [113,114]. OSCC is characterized by relatively high resistance to chemotherapy. It has been proven that resveratrol can act synergistically with conventional drugs, e.g., cisplatin and cetuximab, and even overcome drug resistance [115]. In a cisplatin-resistant OSCC cell line, non-toxic concentrations (50 μM) of resveratrol depleted the migration and invasive capabilities of cancer cells by reducing the mRNA and protein expression of MMP-2 and MMP-9 [114]. 

In lung cancer cells (A549), resveratrol (10 μM, 30 μM) decreased MMP-9 mRNA expression and activity and its promoter activity, without any significant impact on MMP-2. This effect resulted from the suppression of NF-κB and activator protein 1, which are directly involved in MMP-9 transcription [116]. Resveratrol treatment has been found to influence similar processes when administered with gold nanoparticles in breast cancer cells: treatment (10 μM) inhibited MMP-9 at the mRNA and protein levels and reduced its activity by downregulating the NF-κB, PI3 K/Akt, and MAPK/ERK pathways. These effects were accompanied by slightly increased protein expression of TIMP-1 and TIMP-2. Consequently, the migratory capacity and invasive ability of the cells were significantly suppressed [117]. Resveratrol at a concentration of up to 50 μM also inhibited both MMP-2 and MMP-9 in other breast cancer cell lines. Interestingly, in addition to blocking MMPs, it was found to attenuate the metastasis and migration of breast cancer cells by reversing the EMT and interfering with tumor–stromal cross-talk [118,119]. 

Resveratrol (20 μM, 40 μM) also suppressed the migratory and invasive abilities of cervical cancer cells by reducing MMP-2 and MMP-9 protein expression [120]. Interestingly, another stilbene, pterostilbene, was found to exert stronger effects and demonstrate greater bioavailability than resveratrol at the same concentration; it is structurally similar to the latter but with two methoxy groups instead of two hydroxyl groups. However, further research is still needed to better verify its therapeutic potential [120].

In colorectal cancer, resveratrol (5 μM) reduced the MMP-9 protein level by modulating the cellular response to TNF-β and downregulating the NF-κB pathway. It is also able to re-chemosensitize cancer cells to 5-fluorouracil and prevent the expression of EMT markers [121,122]. Also, it was proven that resveratrol (12.5 μM, 25 μM, 50 μM) impaired the expression of EMT-related genes, including MMP-2 and MMP-9, in pancreatic cancer; such inhibition via the suppression of the NF-κB, PI3 K/Akt and uPA pathways reduced cell proliferation, invasion, and metastasis in a concentration-dependent manner [123,124]. 

In HCC, resveratrol (10 μg/mL) limited the expression of MMP-2 and MMP-9 at the mRNA and protein levels and increased TIMP-1 and TIMP-2. Together with the modulation of multiple signaling pathways, this effect lessened cell viability and stimulated apoptosis [125]. Furthermore, resveratrol could enhance the anti-cancer activity of paclitaxel and gemcitabine in HCC and pancreatic cancer [125,126]. Also, resveratrol treatment (25 μM, 50 μM, 100 μM) limited the proliferation, migration, and invasion of neoplastic cells in renal cancer by inhibiting MMP-2 and MMP-9 and stimulating TIMP-1 by modulating the MAPK/ERK and PI3 K/Akt pathways [127]. However, it must be emphasized that these effects may result from the polyphenol regulating histone acetylation in renal carcinoma [128]. Additionally, resveratrol used at different concentrations, detailed in Table 6, has been found to mitigate the adhesive, migratory, and invasive properties of cancer cells by simultaneously downregulating MMP-2 and MMP-9 and upregulating TIMP-1 and TIMP-2 in bladder cancer [129], prostate cancer [130], and thyroid carcinoma [131]. Resveratrol treatment has also demonstrated similar effects in several mesenchymal cancers, e.g., osteosarcoma [132] and chondrosarcoma [133]. In contrast, resveratrol significantly increased MMP-9 activity and expression and improved cell motility in fibrosarcoma, promoting disease progression [134]. 

Resveratrol’s anti-metastatic properties were confirmed in mouse breast cancer 4 T1 cells, where it reduced the activity and expression of MMP-9 in a concentration-dependent manner and mitigated cell adhesion, motility, and invasion [135]. Similar effects were demonstrated in SKBR3/paclitaxel-resistant (SKBR3/PR) breast cancer cells. Free resveratrol and resveratrol–solid lipid nanoparticles, with or without D-α-tocopheryl polyethylene glycol 1000 succinate, were found to inhibit the protein expression of MMP-2 and MMP-9 and to modulate EMT markers, thus attenuating cell migration and invasion [136]. In a model of oral cancer, resveratrol suppressed the metastasis and invasion of H-357 cells by inhibiting the activity of MMP-2 and MMP-9 and numerous inflammation mediators, e.g., NF-κB, COX-2, inducible nitric oxide synthase (iNOS), TLR4, TNF-α, interleukin-1 β (IL-1 β), and interleukin-6 (IL-6). Additionally, resveratrol administration suppressed angiogenesis in the cancer cells, as indicated by the Chick Chorioallantoic Membrane assay [137]. An investigation by Yang et al. [138] found resveratrol to have anti-cancer effects on gastric cancer. In SGC-7901 and HSC-39 cells, the polyphenol reversed IL-6-induced overexpression of MMP-2 and MMP-9. Resveratrol inhibited TGF-β1-induced expression of MMP-2 and MMP-9 in LoVo cells [139]. Wang and co-workers [140] assessed the impact of resveratrol on malignant C6 rat glioma cells. Treatment downregulated multiple signaling pathways and mediators, such as NF-κB, PI3 K/Akt, mTOR, COX-2, EGFR, and VEGF, in a concentration-dependent manner. Immunohistochemical staining confirmed the diminished expression of MMP-9 and NF-κB. A similar experiment, but on glioblastoma-initiating cells (GICs), revealed reduced MMP-2 activity and protein expression, probably due to the downregulation of the NF-κB and PI3 K/Akt/mTOR pathways [141]. Finally, Yang and co-workers [142] evaluated the anti-cancer effect of resveratrol against human osteosarcoma. The treatment of HOS cells caused the concentration-dependent suppression of MMP-2 mRNA and protein expression, enzymatic activity, or promoter activity. Additionally, resveratrol inhibited MMP-2 transcriptional activity by blocking the DNA-binding activity of cAMP response-element binding, a form of transcription factor. Interestingly, TIMP-2 expression was not affected by resveratrol. In addition, resveratrol also modulated some cellular signaling pathways, such as Akt or c-Jun N-terminal kinases; this could be responsible for suppressing MMP-2 and weakening cancer cell motility, adhesion, and invasion. 

Selected studies investigating the beneficial effects of resveratrol in vitro are summarized in Table 10. 

#### 3.5.2. In Vivo Studies 

The anti-cancer properties of resveratrol were demonstrated in an in vivo model of HCC. HCC was chemically induced in male Sprague-Dawley rats using diethylnitrosamine and carbon tetrachloride. The following experimental variants were used: alone in low (300 mg/kg) and high (450 mg/kg) doses for 9 months, pretreated for 4 weeks before the administration of carcinogenic compounds and for 9 months after it, or without the pretreatment for 9 months. The tumor-bearing animals treated with the polyphenol demonstrated significantly lower MMP-2 and MMP-9 serum concentrations compared to the untreated group. Interestingly, the post-treated group presented a more noticeable effect than the pretreated. Also, resveratrol reduced the serum concentrations of sirtuin 1 and VEGF, as well as those of heparanase and elastase, two other enzymes known to degrade the ECM. The histopathological assessment demonstrated that resveratrol partially reversed the morphological changes in the liver induced by carcinogens, indicating the anti-proliferative, anti-metastatic, and anti-angiogenic properties of resveratrol [143]. 

A study performed by Ganapathy et al. [144] found resveratrol to have anti-tumor properties against prostate cancer. A xenograft model was established by injecting PC-3 cells into the right flank of athymic Balb/c nu/nu nude mice. The oral application of resveratrol (30 mg/kg) caused the significant inhibition of MMP-2 and MMP-9 expression, as detected by immunohistochemistry and Western blotting. Also, the growth of tumors and the expression of pro-angiogenic VEGF were limited, confirming the anti-metastatic and anti-angiogenic effects. 

To evaluate the effects of resveratrol on metastasis formation in vivo, breast cancer cells were intravenously injected into the tail veins of five-week-old female Balb/c mice. The polyphenol was orally administered at different doses (100 and 200 mg/kg of body weight). The cancer foci were significantly smaller in the resveratrol groups, possibly due to the decreased activity of MMP-9 in plasma, as confirmed using gelatin zymography [135]. In a study by Wang et al. [136], SKBR3/PR cells were implanted into the right flank of female Balb/c nude mice, which were then intraperitoneally treated with resveratrol (20 mg/kg) five times a day for 21 days. The treatment limited tumor growth and decreased its volume. In a xenograft model of oral cancer, created by injecting H-357 cells into Balb/c mice, resveratrol treatment caused a reduction in tumor volume [137]. 

In a study by Yang et al. [138], resveratrol attenuated the formation of metastases in the lungs of male NOD-SCID mice with HSC-39 cells injected into the tail vein. The results demonstrate the potential of resveratrol to inhibit IL-6-related cancer progression and metastasis. Another study assessed the effect of resveratrol against LoVo colorectal cancer. Briefly, female Balb/c nude mice aged four to six weeks were subjected to orthotopic implantation and tail injection with the cancer cells. Fragments of a tumor developed in one mouse were transplanted into the appendixes of other animals. The mice treated with a three-week intragastric administration of resveratrol at increasing doses (50, 100, and 150 mg/kg) resulted in fewer metastases in the lungs and suppressed local invasion and tumor growth. These effects were dose-dependent [139]. 

In a study by Wang and co-workers [140], C6 cells were stereotactically implanted into the right caudate nucleus of Wistar rats. The oral application of resveratrol prolonged survival time. Interestingly, only small amounts of cancer cells were visible in histopathological examination. Moreover, reduced MMP-9 expression was confirmed in tumor sections by immunohistochemistry. In a similar study, GICs acquired from patients were stereotactically injected into the right corpus striatum of six-week-old male NOD-SCID mice. Resveratrol (10 mg/kg) administered intraperitoneally once a day for four weeks suppressed cancer invasion. Tumors in the resveratrol-treated group were characterized by smoother borders and infiltrated the gray and white matter less clearly than in the control group [141]. In an orthotopic graft model using SCID mice with MNNG/HOS cells injected into the proximal tibia, the oral application of resveratrol resulted in less metastatic foci in the lungs and smaller origin tumors. Immunohistochemical staining also revealed decreased MMP-2 expression in tumor tissue [142]. 

Selected studies investigating the beneficial effects of resveratrol in vivo are summarized in Table 11. 

### 3.6. Human Studies 

Clinical studies assessing the effectiveness and utility of polyphenols in cancer diseases by suppressing MMP expression are ongoing, and their number is limited. We searched for studies using the ClinicalTrials.gov platform. There are many studies that are in the phase of recruiting or have been completed without available results. Nonetheless, most of them focus on the beneficial effects of curcumin on cancer. In a study with patients with advanced pancreatic cancer, a phase II trial (NCT00094445) determined the effect of oral curcumin (8 mg per day for 8 weeks) on six-month survival and response rate and assessed the biological activity in tumor and blood mononuclear cells by examining signaling and apoptotic pathways. However, detailed results were not published. Another study (NCT00113841) was performed to assess clinical tolerance and response to curcumin alone and in combination with Bioperine in subjects with multiple myeloma (MM). Also, the evaluation of the biological effects of curcumin on the expression of NF-κB and related genes in MM was a primary objective. Two grams of the polyphenol was orally used in two divided doses. The percent change in NF-κB protein expression in peripheral blood mononuclear cells was assessed at baseline and after four weeks of treatment. The particular results were not presented. In turn, a phase I/II pilot study conducted on 13 patients with metastatic colorectal cancer analyzed the association between adding genistein to the regimens of FOLFOX or FOLFOX-Avastin and therapy tolerability, progression-free survival, and response rate measured by radiologic RECIST criteria (NCT01985763). Oral genistein at a dose of 60 mg per day for seven days every two weeks was applied. The therapy contributed to a significant percent regression in tumor size, as well as being associated with a good radiological response. Also, genistein may be useful in prostate cancer. A randomized phase II trial (NCT01126879) determined whether treatment with genistein could reduce the number of circulating prostate cancer cells and tumor growth via the downregulation of the expression of factors promoting tumor growth, including MMP-2, in prostate tissue. In this study, genistein was generally applied once daily for three months beginning at least one month prior to radical prostatectomy. Unfortunately, the trial was closed due to low accrual. There are some clinical trials assessing the efficacy of polyphenols for cancer prevention, as well as for improving the safety and effectiveness of chemotherapy (NCT01538316, NCT01496521, NCT06080841, NCT06398405, NCT06015022, NCT02891538, NCT01912820, NCT00027495, NCT01490996); however, their detailed status is unknown, patients are still being recruited, or detailed results have not been posted yet. The number of human studies confirming the safety and utility of polyphenols is insufficient. Moreover, outcomes from existing studies are not always positive. This results from several factors, such as the higher doses of the compounds used in animal studies compared to those applied in humans, among other factors. Large-scale, well-controlled human clinical trials are needed to confirm the health-promoting effects of consuming polyphenols.

## 4. The Mechanisms of MMP-2 and MMP-9 Regulation via Polyphenols 

Many signaling pathways are responsible for the modulation of type IV collagenase expression and activity, which can affect cell growth, survival, migration, invasiveness, or even metastasis formation. The enhanced expression and activity of MMP-2 and MMP-9 are associated with the increased activity of various cellular signaling pathways, such as NF-κB, MAPK, or Akt. Also, it has been found that by suppressing these pathways, polyphenols may inhibit MMP activity [10]. The PI3 K/Akt cellular signaling pathway has a significant role in cell growth and survival, determining the development of potential cell malignancy and invasiveness. Several studies indicate that polyphenols have anti-cancer potential that is realized through the inhibition of MMPs by suppressing the PI3 K/AKT pathway. Resveratrol was found to suppress the activity of the PI3 K/Akt pathway, thus limiting the EMT, cell migration, and metastasis [118]. In addition, EGCG and curcumin appear to restrict the proliferation of bladder cancer and colon cancer, as well as limiting tumor growth and progression, by inhibiting the PI3 K/Akt pathway [145,146]. 

The activation of the MAPK and NF-κB pathways enhances the expression of MMP-2 and MMP-9, thus promoting cell invasion and migration and cancer disease progression [147,148]. However, curcumin treatment inhibited the proliferation and invasion of glioma cells by downregulating the ERK/MAPK pathway [36]. In turn, curcumin suppressed the growth and invasion of human monocytic leukemia cells by reducing MMP-2 and MMP-9 mRNA transcription and protein expression by downregulating the ERK and NF-κB signaling pathways [36]. Also, curcumin is able to reduce the resistance of lung cancer cells to therapy by suppressing NF-κB [149]. Furthermore, other polyphenols described in our article were demonstrated as compounds having the ability to suppress intracellular pathways. Quercetin was found to exert anti-cancer effects, cause cell cycle arrest, leading to cancer cell death, and limit the progression of the disease by downregulating the PI3 K/Akt, MAPK/ERK, JNK, p38, and NF-κB pathways [150,151]. In turn, genistein inactivates MAPK, Akt, NF-κB, and ERK1/2, which cause downstream MMP expression, and, consequently, exerts anti-cancer and anti-angiogenic effects [64]. 

**Figure 2:**
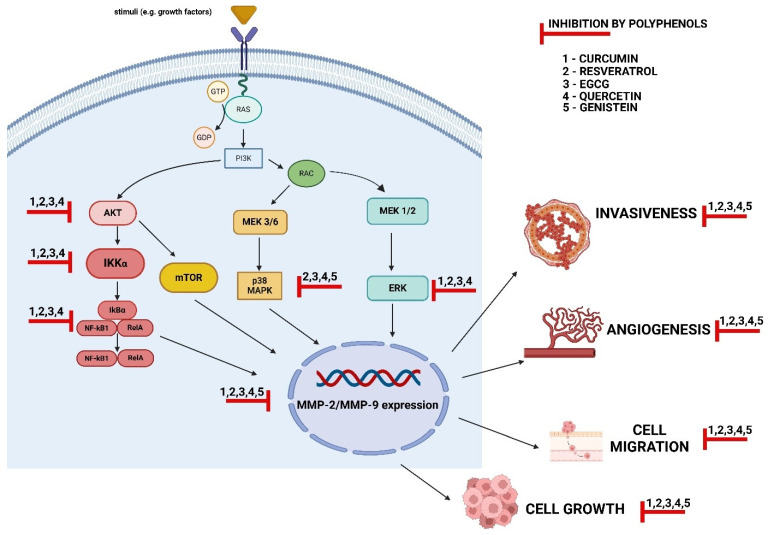
A schematic representation of the PI3 K/AKT, MAPK, and NF-κB pathways involved in the inhibition of expression and activity of MMP-2/9 by polyphenols (created by BioRender). AKT—protein kinase B; ERK—extracellular-signal-regulated kinase; IKK—I kappa B kinase; IKKα—catalytic subunit of IKK; IκBα—inhibitor alpha of nuclear factor-kappa B; GTP—guanosine-5′-triphosphate; MEK—mitogen-activated protein kinase; MMP-2/9—matrix metalloproteinase-2/9 (type IV collagenases); mTOR—mammalian target of rapamycin; NF-κB—nuclear factor-kappa B; PI3 K—phosphatidylinositol 3-kinase; p38 MAPK—p38 mitogen-activated protein kinase; RAS—rat sarcoma, subfamily of small GTPases; RelA—subunit of NF-κB; EGCG—epigallocatechin-3-gallate.

The mechanisms by which polyphenols suppress type IV collagenases via cellular signaling pathways are summarized in Figure 2.

## 5. Summary and Conclusions

A considerable body of research has accumulated regarding the effects of individual polyphenols, such as curcumin, resveratrol, EGCG, genistein, and quercetin, as treatments for cancer. They are believed to exert their anti-invasive and anti-metastatic activities by downregulating the expression and activity of type IV collagenases and by suppressing cellular signaling pathways, particularly NF-κB and MAPK. Pre-clinical studies employing both in vitro and animal models suggest that they mostly act by inhibiting MMP-2 and MMP-9 or their related pathways, indicating that polyphenolic compounds have considerable potential against the development and progression of cancers. 

The preventive and therapeutic efficacy of polyphenols as modulators of gelatinases in cancers remains controversial due to some limitations in the research described. Most of the studies described herein focus on the use of natural polyphenolic compounds in pre-clinical studies. These findings show that polyphenols are able to prevent cancer development by inhibiting MMP-2/MMP-9 and suppressing various signaling pathways. Unfortunately, the number of human studies is small, and those available were restricted to small participant groups, short-term administration, and usually one dose without a placebo group. The results of this review hint at the potential benefits associated with polyphenolic compounds, but more studies on patients are necessary to establish the efficacy and safety of polyphenols as inhibitors of MMP-2 and MMP-9. The large variation in the bioavailability of ingested compounds and incomplete information in food composition databases are only some of the main challenges related to their use. The effects of single polyphenolic compounds applied in pharmacological doses in epidemiological studies and animal models may, however, not be transferable to polyphenol levels achievable with normal human diets. 

The most recent data show that polyphenols are characterized by unsatisfactory bioavailability and bioaccessibility, which may significantly limit the efficacy of their oral administration [152]. Therefore, there is a need to create new formulations or delivery systems with greater bioavailability. Nevertheless, taking into account their properties and metabolism, polyphenols offer promise as chemopreventive agents rather than as therapeutic ones. Further studies, primarily involving human participants in clinical trials, are necessary to confirm the anti-cancer properties of polyphenols as agents with chemopreventive activity.

## Figures and Tables

**Table 1:** Overview of polyphenols described in this review.

Polyphenol	Class of Compounds	Main Pro-Health Activities	Main Food Sources of Polyphenol
Curcumin (1,7-bis(4-hydroxy-3-methoxyphenyl)-1,6-heptadiene-3,5-dione)	Curcuminoids	Anti-cancer, anti-inflammatory, anti-migratory, anti-metastatic, anti-aging	The rhizome of *Curcuma longa*
EGCG (2 R,3 R)-3′,4′,5,5′,7-pentahydroxyflavan-3-yl 3,4,5-trihydroxybenzoate)	Flavonoids: flavan-3-ols	Anti-oxidative, anti-cancer, anti-inflammatory	Green tea
Genistein (4′,5,7-trihydroxyisoflavone)	Flavonoids: isoflavones	Anti-cancer, anti-oxidant, anti-inflammatory, anti-angiogenic, proapoptotic, neuroprotective	Fermented soya (miso and natto), soy nuts, soy powder, soy milk, tofu
Quercetin (3,3′,4′,5,7-pentahydroxyflavone)	Flavonoids: flavonols	Anti-proliferative, anti-inflammatory, anti-carcinogenic, anti-oxidant, anti-bacterial, anti-viral	Onions, capers, green tea, apples, broccoli, red leaf lettuce, cherries, ginkgo, American elderberry, hypericum
Resveratrol (3,5,4′-trihydroxystilbene)	Stilbenes	Cardioprotective, anti-oxidative, anti-inflammatory, neuroprotective, anti-diabetic, anti-cancer	Grapes, red wine, chocolate, berries, mulberries, peanuts

**Table 2:** Overview of in vitro studies related to curcumin.

Polyphenol	Cell Line	Concentration/ Duration	Anti-Cancer Effects	Type IV Collagenase-Mediated Mechanisms	Reference
Curcumin [1,7-bis(4-hydroxy-3-methoxyphenyl)-1,6-heptadiene-3,5-dione]	N18	7,5, 15 μM for 6, 12, 24, 48 h	↓cell migration ↓cell invasion	↓MMP-2 mRNA, protein ↓MMP-9 protein ↓NF-κB p65 ↓ERK-1/-2	[31]
	MDA-MB-231	10, 20, 40 μM for 24, 48, 72 h	not specified	↓MMP-2, -9 mRNA ↑TIMP-1, -2, -3, -4 mRNA	[29]
	Ishikawa Hec-1 B Primary endometrial adenocarcinoma cells	6 μM for 24, 30, 36, 42, 48 h	↓proliferation ↓cell migration	↓MMP-2 protein ↓MMP-9 protein	[32]
	HepG2 PLC/PRF/5	2,5, 5, 10 μM for 12, 24, 48 h with or without 2,5, 5, 10 mM metformin	↓angiogenesis ↓cell migration ↓cell invasion ↓proliferation ↑apoptosis	↓MMP-2, -9 protein, activity ↓PI3 K protein ↓pAkt protein ↓pmTOR protein	[35]
	WT Primary cells WT-1, WT-2, WT-3	10, 20 mM for 6, 12, 18, 24 h	↓cell migration ↓cell invasion ↓proliferation ↑apoptosis	↓MMP-2, -9 mRNA, protein	[34]
	LN229 U87 MG	8, 16, 24 μM for 24, 48, 72 h with or without 0,1, 1, 10 μM of norepinephrine	↓cell migration ↓cell invasion ↓proliferation ↑apoptosis	↓MMP-2, -9 mRNA, protein, activity ↓pERK-1/-2 protein	[36]
	HepG2 SK-Hep-1	20, 40, 60 μM for 24 h	↓cell migration ↓cell invasion ↓proliferation	↓MMP-2, -9 protein	[33]

Legend: ↑ activation or increase; ↓ inhibition or decrease.

**Table 3:** Overview of in vivo studies related to curcumin.

Polyphenol	Animal Model	Dose/ Duration	Anti-Tumor Effects	Type IV Collagenase-Mediated Mechanisms	Reference
Curcumin [1,7-bis(4-hydroxy-3-methoxyphenyl)-1,6-heptadiene-3,5-dione]	NOD-SCID mice injected with Ishikawa	50 mg/kg daily intraperitoneally for 31 days	↓tumor volume	↓MMP-2 protein ↓MMP-9 protein	[32]
	Balb/c mice injected with C26	5 mg/kg curcumin with or without 2.5 mg/kg doxorubicin (in free form or long-circulating liposomes, LCLs) intravenously at days 7 and 10 after inoculation	↓tumor volume	↔MMP-2 activity ↔MMP-9 activity (free curcumin) ↓MMP-9 activity (LCL–curcumin, curcumin with doxorubicin) ↓pNF-κB p65 protein	[40]
	C57 B16 mice injected with B16 F10	15 mg/kg of free curcumin or 30 mg/kg of nano-encapsulated curcumin daily intraperitoneally for 12 days (with or without identical doses of chrysin)	↓tumor volume	↓MMP-2, -9 mRNA ↑TIMP-1, -2 mRNA	[39]
	Balb/c-nu mice injected with HepG2	60 mg/kg daily intraperitoneally for 21 days with or without 150 mg/kg metformin orally	↓tumor volume	↓MMP-2, -9 protein ↓PI3 K protein ↓pAkt protein ↓pmTOR protein	[35]
	Nude mice injected with primary WT-1 and WT-3	20 mg/mL of curcumin in corn oil for 21 days	↓tumor weight ↓tumor volume ↑apoptosis	not specified	[34]
	SCID mice injected with SHI-1	15, 30 mg/kg daily intraperitoneally for 15 days	↓tumor weight ↓tumor volume ↑apoptosis	↓MMP-2, -9 mRNA, protein ↑pp38 protein ↓pERK-1/-2 protein ↓pNF-κB p65 protein	[37]
	Balb/c nu/nu mice injected with LN229	60 mg/kg daily intraperitoneally for 4 weeks, accompanied or not by restraint stress for 8 h per day	↓tumor volume	↓MMP-2, -9 protein	[36]
	Balb/c-nu mice injected with HepG2	0.2 mL of 20, 40, 60 μM curcumin daily intraperitoneally for 10 days	↓tumor volume ↓metastatic incidence in lungs	↓MMP-2, -9 protein	[33]

Legend: ↑ activation or increase; ↓ inhibition or decrease; ↔ no impact.

**Table 4:** Overview of in vitro studies related to EGCG.

Polyphenol	Cell Line	Concentration/ Duration	Anti-Cancer Effects	Type IV Collagenase-Mediated Mechanisms	Reference
EGCG [(2 R,3 R)-3′,4′,5,5′,7-pentahydroxyflavan-3-yl 3,4,5-trihydroxybenzoate]	DU-145	2, 5, 10, 20, 40 μg/mL for 24 h	not specified	↓MMP-2, -9 secretion, activity ↓NF-κB ↓ERK-1/-2 ↓p38	[46]
	786-O	10, 50, 100, 200 μg/mL for 24 h with or without PMA treatment	not specified	↓MMP-2, -9 activity	[53]
	HeLa DoTc2-4510 SK-OV-3	10, 25, 50, 100 μM for 24 h with or without PMA treatment	not specified	↓MMP-2 activity (HeLa, SK-OV-3) ↓MMP-9 activity (HeLa, DoTc2-4510)	[52]
	SCC-9	5, 10, 15, 20 μM for 24 h with or without PMA treatment	↓cell migration ↓cell invasion	↓MMP-2 mRNA, protein, activity ↓MMP-9 activity ↑TIMP-2 mRNA, protein ↓NF-κB protein	[62]
	CL1-5	5, 10, 20, 30, 40, 50 μM for 9, 12, 16, 24 h	↓cell migration ↓cell invasion	↓MMP-2 mRNA, protein, activity, promoter activity ↓MMP-9 mRNA, protein, activity ↓NF-κB nuclear translocation	[60]
	SW-1353, HT-1080, SW-872, SW-982	10, 25, 50, 100 μM for 24 h with or without PMA treatment	not specified	↓MMP-2, -9 activity	[51]
	U2 OS RD	10, 25, 50, 100 μM for 24 h with or without PMA treatment	not specified	↓MMP-2 activity ↓MMP-9 activity	[50]
	TW01 (EBV-negative) NA (EBV-positive, reinfected TW01)	10, 20, 30, 50 μM for 9, 12, 24, 48 h (cells) 1, 2.5, 5, 10, 25 μM for 7 days (spheroid)	↓spheroid formation ↓cell migration ↓cell invasion ↓proliferation	↓MMP-2 mRNA, activity ↓MMP-9 activity ↓pERK-1/-2 protein	[61]
	786-O ACHN	10, 20, 40 μg/mL for 24 h	↓cell migration ↓cell invasion	↓MMP-2, -9 activity, protein	[55]
	HuCC-T1	0.1, 0.5, 1, 5, 10, 20, 50 μg/mL for 24 h	↓cell migration ↓cell invasion ↑apoptosis	↓MMP-2 activity ↓MMP-9 activity	[56]
	SW780	12.5, 25, 50, 100 μM for 24 h	↓cell invasion ↓cell migration ↓proliferation ↑apoptosis	↓MMP-9 mRNA, protein ↓pNF-κB p65 protein ↓NF-κB mRNA	[57]
	A-2058	10, 25, 50, 100 μM for 24 h	not specified	↓MMP-2, -9 activity	[48]
	FaDu SCC-25	10, 25, 50, 100 μM for 24 h with or without PMA treatment	not specified	↓MMP-2, -9 activity	[49]
	NPC-39 HONE-1 NPC-BM	6, 12, 25, 50 μM for 12, 24 h	↓cell migration ↓cell invasion	↓MMP-2 activity, protein	[54]
	A549 H1299	20, 80, 160 μM for 24, 48 h or 20 μM with 0,625, 1.25, 2.5, 5 μM of BAY11-7082 for 24, 48 h	↓proliferation ↑apoptosis ↓cell invasion ↓cell migration	↓MMP-2 mRNA ↓NF-κB, pNF-κB mRNA, protein	[59]
	A549 H1299	12.5, 25 μM for 48 h (free EGCG or PLGA-encapsulated EGCG)	↓proliferation ↑apoptosis	↓MMP-2 mRNA ↓NF-κB, pNF-κB protein	[58]

Legend: ↑ activation or increase; ↓ inhibition or decrease.

**Table 5:** Overview of in vivo studies related to EGCG.

Polyphenol	Animal Model	Dose/ Duration	Anti-Tumor Effects	Type IV Collagenase-Mediated Mechanisms	Reference
EGCG [(2 R,3 R)-3′,4′,5,5′,7-pentahydroxyflavan-3-yl 3,4,5-trihydroxybenzoate]	Balb/c nu/nu mice injected with SCC-9	10, 20 mg/kg daily through oral gavage for 45 days	↓tumor weight ↓tumor volume	not specified	[62]
	Balb/c nude mice injected with CL1-5	50 mg/kg intraperitoneally twice a week for 6 weeks	↓metastatic incidence in lungs	not specified	[60]
	Balb/c nu/nu mice injected with PANC-1	60, 80, 100 mg/kg daily by gavage, 5 days a week for 28 days	↓angiogenesis ↑apoptosis ratio ↓pancreas weight	↓MMP-2 mRNA ↓pAkt protein ↓pPI3 K protein ↓pERK protein	[63]
	SCID mice injected with NA (EBV-positive)	30 mg/kg every day or 50 mg/kg every two days, by oral gavage for 8 weeks	↓tumor volume	not specified	[61]
	Balb/c mice injected with HuCC-T1	20 mg/kg once subcutaneously beside tumor when it reached 4–5 mm in diameter	↓tumor volume	↓MMP-2, -9 protein	[56]
	Balb/c mice injected with SW780	25, 50, 100 mg/kg daily intraperitoneally for 3 weeks	↓tumor weight ↓tumor volume	↓MMP-9 mRNA, protein ↓pNF-κB p65 protein ↓NF-κB mRNA	[57]
	Balb/c athymic nude mice injected with A549	20 mg/kg with or without 10 mg/kg BAY11-7082, intraperitoneally for 21 days	↓tumor weight ↓tumor volume	↓pNF-κB protein	[59]
	Balb/c athymic nude mice injected with patient-derived lung cancer samples	10 mg/kg (free EGCG) or 5 mg/kg (PLGA-encapsulated EGCG) daily intraperitoneally for a month	↓tumor weight ↓tumor volume	↓pNF-κB protein	[58]

Legend: ↑ activation or increase; ↓ inhibition or decrease.

**Table 6:** Overview of in vitro studies related to genistein.

Polyphenol	Cell Line	Concentration/ Duration	Anti-Cancer Effects	Type IV Collagenase-Mediated Mechanisms	Reference
Genistein (4′,5,7-trihydroxyisoflavone)	TGF-β1-induced Panc-1	1, 25, 50 μM for 24, 48 h	↓cell migration ↓cell invasion	↓MMP-2 mRNA, activity ↔MMP-9 mRNA, activity ↓uPA mRNA, protein ↓p-p38 MAPK	[75]
	HeLa	5, 25, 100 μM for 6, 24, 48 h	↓cell migration ↑apoptosis	↓MMP-9 mRNA ↑TIMP-1 mRNA	[70]
	A549	25, 50, 75, 100 μM for 24, 48 h	↓proliferation	↓MMP-2 mRNA, activity ↔MMP-9 mRNA, activity	[74]
	HCCLM3	40 μM for 24, 48 h with or without 20 μM of cisplatin	↓proliferation	↓MMP-2 protein	[77]
	518 A2	25, 50 μM for 6, 24, 48 h Free genistein and Cu(II)–genistein complex	↓cell migration ↓cell invasion ↓proliferation	↓MMP-2, -9 secretion, activity	[73]
	HCT116 SW620 HT29	10, 50 μM for 24, 48 h	↓cell migration ↓cell invasion ↓proliferation	↓MMP-2 mRNA, protein	[76]
	HT29	10, 30, 50, 70 μM for 12, 24, 48 h	↓cell migration ↓proliferation ↑apoptosis	↓MMP-2 activity ↓p38 MAPK mRNA ↓p-p38 MAPK	[68]
	Mia-PaCa2	5, 10, 20, 40 μM for 5, 10, 12, 20, 24, 48 h	↓cell migration ↑apoptosis	↓MMP-2, -9 protein	[71]
	HT29	10, 20, 60 μM for 24, 48, 72 h	↓cell migration ↓cell invasion	↓MMP-2, -9 mRNA, protein ↑TIMP-1 mRNA, protein	[69]
	PC3	10, 30, 50, 70 μM for 6, 12, 24 h	↓cell migration ↓proliferation ↑apoptosis	↓MMP-2 activity ↓p38 MAPK mRNA ↓p-p38 MAPK	[67]

Legend: ↑ activation or increase; ↓ inhibition or decrease; ↔ no impact.

**Table 7:** Overview of in vivo studies related to genistein.

Polyphenol	Animal Model	Dose/ Duration	Anti-Tumor Effects	Type IV Collagenase-Mediated Mechanisms	Reference
Genistein (4′,5,7-trihydroxyisoflavone)	Balb/c nu/nu mice injected with HCCLM3; partial hepatectomy 10 days after inoculation	2 mg/kg daily for 4 weeks or 2 mg/kg cisplatin for 7 days or combined therapy of above, intraperitoneally, beginning 3 days after liver lobe resection	↓recurrent tumor volume ↓metastatic incidence in lungs	↓MMP-2 mRNA, protein	[77]
	Balb/cA Jcl-nu and C3 H mice injected with LM8; LM8 previously treated with 50 μM genistein for 3 days	Laboratory chow and water for 25 days (nude) or 36 days (C3 H), no genistein administration	↓tumor weight ↓engraftment rate ↓metastatic incidence in liver ↓metastatic incidence in lungs (by 100%)	↓MMP-2 protein	[78]
	Balb/c athymic mice injected with HCT116-LUC	25, 75 mg/kg daily orally 5 days a week, for 5 weeks	↓tumor weight ↓tumor volume ↓metastatic incidence in liver ↓metastatic incidence in lungs	↓MMP-2 protein	[76]

Legend: ↓ inhibition or decrease.

**Table 8:** Overview of in vitro studies related to quercetin.

Polyphenol	Cell Line	Concentration/ Duration	Anti-Cancer Effects	Type IV Collagenase-Mediated Mechanisms	Reference
Quercetin (3,3′,4′,5,7-pentahydroxyflavone)	A375 A2058 B16 F10	10, 20, 40, 60 µM for 16, 24 h or 60 µM for 3, 6, 12, 24 h or 20, 40, 60, 80 µM for 24 h	↓cell migration ↓cell invasion ↓proliferation ↑apoptosis	↓MMP-2, -9 mRNA, activity ↑pERK, ↑pAkt protein	[105]
	MCF-7 MDA-MB-231	50, 100 µM for 24 h (quercetin or gold-nanoparticle-conjugated quercetin)	↓cell migration ↓cell invasion ↓proliferation ↓angiogenesis	↓MMP-2, -9 protein ↓pPI3 K, ↓Akt, ↓pAkt	[100]
	CT26 MC38 CCD-18 Co	10, 25, 50, 100 µM for 24 h or 50 µM for 3, 6, 9, 12 h or 50 µM for 15, 30, 60 min or 0.1, 1, 10 µM for 24 h	↓cell migration ↓cell invasion ↓proliferation ↑apoptosis	↓MMP-2, -9 mRNA, activity ↑TIMP-1, -2 mRNA ↑pERK, pp38 protein	[102]
	A549 H1975 HCC827	10, 25, 50 µM for 24 h or 50 µM for 10, 30 min, 6, 24 h or 50 µM for 1, 2, 4, 8, 24 h	↓cell migration ↓cell invasion	↓MMP-2 protein ↓pAkt	[101]
	HOS MG63	25, 50, 100 µM for 6, 12, 24 h	↓cell migration ↓cell invasion	↓MMP-2, -9 mRNA, protein	[106]
	U251	10, 20, 30, 40 μg/mL for 24, 48 h	↓cell migration ↓cell invasion ↓proliferation ↑apoptosis	↓MMP-2, -9 protein	[85]
	U251	10 μg/mL for 24, 48 h	↓cell migration ↓cell invasion	↓MMP-2, -9 protein	[86]
	MCF-7	25, 50, 100, 200 µM for 4, 5, 24, 72 h (quercetin) 0.7, 1.4, 2.8, 5.6 mg/mL for 4, 5, 24, 72 h (hyaluronic acid nanohydrogel of quercetin) with or without 1, 10 nM of Everolimus	↓proliferation ↑apoptosis	↓MMP-2, -9 protein	[95]
	IL-6-induced PATU-8988	20, 40, 80 µM for 24, 48 h	↓cell migration ↓cell invasion	↓MMP-2 mRNA, protein	[92]
	MCF-7 MDA-MB-231	20, 30, 40, 60, 80, 100 µM for 6, 24 h	↓cell migration ↓cell invasion	↓MMP-2, -9 protein ↓pAkt protein ↓pmTOR protein	[99]
	BGC823 AGS	10 µM for 72 h	↓cell migration ↓cell invasion ↓proliferation	↓MMP-2, -9 activity ↓pNF-κB p65 protein ↓pERK-1/-2 protein	[91]
	Nickel-induced A549	2, 5 µM for 24 h 2, 5 µM for 4 h before Nickel (1 mM) for 5–10 min or 1, 1.5, 12 h	↓cell migration ↓cell invasion	↓MMP-2 activity ↓MMP-9 activity, protein ↓NF-κB p65 protein ↓IKKβ protein ↓I-κBα protein	[84]
	U2 OS Saos-2	20, 40, 60, 80, 100 µM for 48 h	↓cell migration ↓cell invasion ↓proliferation ↓cell adhesion ↑apoptosis	↓MMP-2, -9 mRNA ↑TIMP-1, -2 mRNA	[89]
	HSC-6 SCC-9	50 µM for 24, 48 h	↓cell migration ↓cell invasion ↓proliferation	↓MMP-2, -9 protein	[88]
	MCF-7	80 µM for 24 h with or without 5 µM of lonidamine	↓proliferation ↑apoptosis	↓MMP-2, -9 mRNA	[97]
	MCF-7	5, 100 µM for 12, 24, 48 h with or without 5 µM of tamoxifen	↑proliferation (5 µM) ↓proliferation (100 µM) ↑cell migration (5 µM) ↓cell migration (100 µM) ↑cell invasion (5 µM) ↓cell invasion (100 µM) ↓apoptosis (5 µM) ↑apoptosis (100 µM)	↑MMP-2 mRNA (5 µM) ↓MMP-2 mRNA (100 µM) ↑MMP-9 mRNA (5 µM) ↓MMP-9 mRNA (100 µM)	[94]
	PA-1	50, 75 µM for 24 h	↓cell migration ↓cell adhesion ↓proliferation	↓MMP-2 mRNA, protein, activity ↓MMP-9 mRNA, protein, activity ↓PI3 K/pPI3 K mRNA, protein ↓Akt/pAkt mRNA, protein ↓mTOR/pmTOR mRNA, protein ↓ERK-1/-2 protein	[93]
	Eca109	5, 10 μg/mL for 8, 12, 24 h	↓cell migration ↓cell invasion ↓proliferation	↓MMP-2, -9 protein	[90]
	MDA-MB-231	50 µM for 24, 48 h with or without 32 nM of doxorubicin	↓cell migration ↓proliferation	↓MMP-2, -9 mRNA	[96]
	A549	20, 40 µM for 24 h	↓proliferation ↓cell migration ↓cell invasion	↓MMP-2, -9 protein ↑TIMP-2 protein ↓pAkt ↓NF-κB	[83]
	H22 HepG2	25, 50, 100 µM for 24, 48, 72 h	↓proliferation ↓cell migration ↓cell invasion ↑autophagy	↓MMP-2, -9 protein ↓pNF-κB p65, ↓pIκBα protein	[103]
	HSC-3 Erlotinib-resistant ERL-R5, ERL-R10	5 µM for 24 h or 5, 10 µM with 5 µM of erlotinib	↓proliferation ↓cell migration ↓cell invasion ↓spheroid formation ↑apoptosis	↓MMP-2, -9 protein	[104]
	ZR-75-1 MCF-7 T47 D MDA-MB-231	2, 5 µM for 2, 10, 12, 24 h	↓proliferation ↓cell migration ↓cell invasion ↓cell adhesion ↑apoptosis	↓MMP-2, -9 protein, activity ↑TIMP-1, -2 protein	[98]

Legend: ↑ activation or increase; ↓ inhibition or decrease.

**Table 9:** Overview of in vivo studies related to quercetin.

Polyphenol	Animal Model	Dose/ Duration	Anti-Tumor Effects	Type IV Collagenase-Mediated Mechanisms	Reference
Quercetin (3,3′,4′,5,7-pentahydroxyflavone)	C57 BL6 mice injected with B16 F10	15 mg/kg daily thrice a week into the peripheral sites of tumors for 3 weeks or 7.5 mg/kg quercetin with 1.75 mg/kg sulforaphane or 15 mg/kg quercetin with 3.5 mg/kg sulforaphane, the same way	↓tumor weight ↓tumor volume	↓MMP-9 protein, activity	[107]
	Balb/c nu/nu mice injected with A375 (xenograft) C57 BL/6 mice injected with B16 F10 (metastasis model)	100 mg/kg daily intragastrically for 21 days (xenograft) 100 mg/kg daily intragastrically, 1 day before cell injection and for next 24 days (metastatic model)	↓tumor weight ↓tumor volume ↓metastatic incidence in lungs	not specified	[105]
	Sprague-Dawley rats stimulated by DMBA	25 mg/kg free quercetin or 25 mg/kg gold-nanoparticle-conjugated quercetin by daily intratumoral injections for 8 days	↓tumor weight ↓tumor volume ↑normal breast tissue architecture	not specified	[100]
	Balb/c mice injected with CT26	10, 50 mg/kg intraperitoneally 2 h prior to cell injection and then once every 2 days for 14 days	↓lung weight ↓metastatic incidence in lungs	not specified	[102]
	SCID mice injected with A549 pretreated with quercetin for 24 h (metastatic model) or not (xenograft)	500 mg/kg daily intraperitoneally for 5 weeks (xenograft model)	↑survival time ↓metastatic incidence in distant organs (xenograft) ↓metastatic incidence in bones ↓metastatic incidence in lungs (metastatic model)	not specified	[101]
	Balb/c nu/nu mice injected with stably transfected HOS	25, 50, 100 mg/kg intraperitoneally, twice daily for a month	↓metastatic incidence in lungs	not specified	[106]
	Balb/c nude mice injected with MCF-7	50 mg/kg, intraperitoneally, twice daily for 25 days	↓tumor volume	↓pAkt protein	[99]
	Balb/c mice injected with H22	25, 50, 100 mg/kg, by gavage, once a day for 21 days	↓tumor weight ↓tumor volume	not specified	[103]
	Nude mice injected with erlotinib-resistant HSC-3, ERL-R5	2, 10 mg/kg, intraperitoneally, daily for 18 days	↓tumor weight ↓tumor volume	not specified	[104]
	Balb/c nude mice injected with ZR-75-1 and MCF-7	20, 40 mg/kg daily, intraperitoneally for 28 days	↓tumor weight ↓tumor volume	not specified	[98]

Legend: ↑ activation or increase; ↓ inhibition or decrease.

**Table 10:** Overview of in vitro studies related to resveratrol.

Polyphenol	Cell Line	Concentration/ Duration	Anti-Cancer Effects	Type IV Collagenase-Mediated Mechanisms	Reference
Resveratrol (3,5,4′-trihydroxystilbene)	PMA-induced A549 HeLa	10, 30 µM for 24 h	↓proliferation ↓cell migration ↓cell invasion	↔MMP-2 mRNA, activity ↓MMP-9 mRNA, activity ↓NF-κB activity	[116]
	4 T1	10, 20, 30 μM for 12, 16, 24, 48 h	↓cell adhesion ↓cell migration ↓cell invasion	↓MMP-9 mRNA, activity	[135]
	HT1080	50 μM for 10, 30 min, 1, 3, 6, 12, 24 h or 10, 20, 30, 40, 50 μM for 24 h or 20, 30, 50 μM for 24, 48 h	↓proliferation ↑cell migration	↑MMP-9 protein, activity ↓pp38 ↓pAkt	[134]
	BxPC-3 Panc-1	12.5, 25, 50 μM for 24, 48 h	↓proliferation ↓cell migration ↓cell invasion	↓MMP-2, -9 mRNA, protein ↓pNF-κB protein ↓pAkt protein	[123]
	HTB94	50 μM for 10, 30 min, 1, 3, 6, 12, 24 h or 10, 20, 30, 40, 50 μM for 24 h	↓proliferation	↓MMP-2, -9 protein, activity ↑pp38	[133]
	TGF-β1-induced LoVo	6, 12 μM for 24, 48 h	↓proliferation ↓cell migration ↓cell invasion	↓MMP-2, -9 protein	[139]
	Glioblastoma-initiating cells (GICs) derived from patients	5, 10, 20 μM for 24, 48 h	↓proliferation ↓cell adhesion ↓cell migration ↓cell invasion	↓MMP-2 protein, activity ↓NF-κB p65 nuclear translocation ↑I-κBα, ↓pI-κBα protein ↑IKKα/β, ↓pIKKα/β protein ↓pAkt, ↓pmTOR protein	[141]
	TPA-induced SCC-9	25, 50, 75, 100 μM for 24 h	↓proliferation ↓cell migration ↓cell invasion	↔MMP-2 activity ↓MMP-9 mRNA, protein, activity ↓pERK protein	[113]
	C6	50, 100, 150 μM for 24, 48 h	↓proliferation ↑apoptosis	↓MMP-9 protein ↓PI3 K, pAkt, mTOR, STAT3 protein ↓NF-κB protein	[140]
	HOS MNNG/HOS 143 B	25, 50, 75, 100 μM for 12, 24, 48 h or 100 μM for 2, 4 h	↓proliferation ↓cell adhesion ↓cell migration ↓cell invasion	↓MMP-2 mRNA, protein, activity, promoter activity ↔TIMP-2 protein ↓pp38, pAkt protein ↑pERK protein	[142]
	SW579	10 μg/mL for 24 h with 100, 200 μg/mL of lentinan	↓proliferation ↑apoptosis	↓MMP-2, -9 mRNA ↑TIMP-1, -2 mRNA ↓NF-κB mRNA ↑I-κBα mRNA	[131]
	Hypoxia-induced BxPC-3 Panc-1	12.5, 25, 50 μM for 24, 48 h	↓proliferation ↓cell migration ↓cell invasion	↓MMP-2 mRNA, protein	[124]
	TPA-stimulated MCF-7	10 μM for 1, 24 h (resveratrol) 1, 5, 10 μM for 1, 4, 12, 24 h (gold-conjugated resveratrol nanoparticles)	↓proliferation ↓cell migration ↓cell invasion	↓MMP-2 protein ↓MMP-9 mRNA, protein, activity, secretion ↑TIMP-1, -2 protein ↓NF-κB p65/pNF-κB p65 protein ↓pAkt, pERK protein	[117]
	U87 MG T98 G U251	40 μM for 24, 48 h	↓proliferation ↓cell migration ↓cell invasion	↓MMP-2 protein, activity	[112]
	T24	10, 25, 50 μM for 6, 12, 24 h	↓cell migration ↓cell invasion	↓MMP-2, -9 protein ↓pERK-1/-2 protein	[129]
	SW480 HCT116	5 μM for 12, 24 h or 10, 28 days	↓proliferation ↓cell invasion (in alginate culture) ↑apoptosis	↓MMP-9 protein ↓pNF-κB p65 protein ↓pNF-κB p50 protein	[121]
	HepG2	10 μg/mL for 24 h with 5, 10 μg/mL of paclitaxel	↑apoptosis	↓MMP-2, -9 mRNA, protein ↑TIMP-1, -2 mRNA, protein ↓NF-κB mRNA, protein ↑I-κBα mRNA, protein	[125]
	U2 OS	6, 12 μg/mL for 24, 48 h	↓proliferation ↓cell migration ↓cell invasion ↑apoptosis	↓MMP-2, -9 protein	[132]
	HCT116 5-Fluorouracil-resistant HCT116 R	5 μM for 24, 72 h or 10 days	↓proliferation ↓cell invasion (in alginate culture) ↑apoptosis	↓MMP-9 protein ↓pNF-κB p65 protein	[122]
	Cancer-associated fibroblasts (CAF)-induced MCF-7 MDA-MB-231	50 μM for 24, 36, 48 h	↓proliferation ↓cell migration ↓cell invasion	↓MMP-2, -9 mRNA ↓pAkt	[119]
	IL-6-induced SGC-7901 HSC-39	10, 20 μM for 48 h	↓proliferation ↓cell invasion	↓MMP-2, -9 activity ↓pERK protein	[138]
	ACHN A498	25, 50, 100 μM for 24, 48 h	↓proliferation ↓cell migration ↓cell invasion	↓MMP-2, -9 protein ↑TIMP-1 protein ↓pAkt, ERK-1/-2, pERK-1/-2	[127]
	TGF-1 β-induced MDA-MB-231 MDA-MB-436 MDA-MB-453 BT549	12.5, 25, 50 μM for 24 h	↓proliferation ↓cell migration ↓cell invasion	↓MMP-2 protein, secretion (MDA-MB-231) ↓MMP-9 protein, secretion (MDA-MB-231) ↓pAkt, pPI3 K	[118]
	HeLa Cancer Stem Cells (CSCs)	10, 20, 40 μM for 24, 48 h	↓proliferation ↑cell migration (slightly, after 24 h) ↓cell migration (after 48 h) ↓cell invasion ↑apoptosis	↓MMP-2, -9 protein	[120]
	PC3 DU145 LNCap	150 μM for 12, 24, 48 h	↓cell migration ↓cell invasion	↓MMP-2, -9 protein ↑TIMP-2, -3 protein ↓TIMP-2, -3 methylation level	[130]
	Cisplatin-resistant CAL27	25, 50, 75 μM for 6, 12, 18, 24 h	↓proliferation ↓cell migration ↓cell invasion	↓MMP-2, -9 protein ↓pERK, pp38 protein	[114]
	H-357	1, 3.5, 5 μM for 48 h (resveratrol nanoparticles)	↓angiogenesis ↓proliferation ↓cell invasion	↓MMP-2, -9 activity ↓NF-κB, IKKα protein ↓Akt, MAPK protein	[137]
	Paclitaxel-resistant SKBR3/PR	30 μM for 24, 48 h (free resveratrol) or equivalent dose (resveratrol–solid lipid nanoparticles with or without TPGS)	↓proliferation ↓cell migration ↓cell invasion ↑apoptosis	↓MMP-2, -9 protein	[136]

Legend: ↑ activation or increase; ↓ inhibition or decrease; ↔ no impact.

**Table 11:** Overview of in vivo studies related to resveratrol.

Polyphenol	Animal Model	Dose/ Duration	Anti-Tumor Effects	Type IV Collagenase-Mediated Mechanisms	Reference
Resveratrol (3,5,4′-trihydroxystilbene)	Balb c nu/nu mice injected with PC-3	30 mg/kg through gavage thrice a week, with or without 15 mg/kg TRAIL intravenously on days 1, 7, 14, 21	↓angiogenesis ↓tumor volume	↓MMP-2, -9 protein	[144]
	Balb/c mice injected with 4 T1	100, 200 mg/kg orally, daily, for 21 days	↓metastatic incidence in lungs	↓MMP-9 activity (in plasma)	[135]
	Sprague-Dawley rats stimulated by DENA and CCl4	diet containing resveratrol 300, 450 mg/kg for 9 months; 300, 450 mg/kg for 4 weeks as pretreatment and for 9 months as post-treatment	↑normal hepatic tissue architecture (post-treatment group)	↓MMP-2, -9 serum concentration	[143]
	Balb/c nude mice injected with LoVo	50, 100, 150 mg/kg via intragastric administration daily for 3 weeks	↓metastatic incidence in lungs (metastatic model and xenograft) ↓metastatic incidence in liver (xenograft) ↓tumor weight (xenograft)	not specified	[139]
	NOD-SCID mice injected with glioblastoma-initiating cells (GICs) derived from patients	10 mg/kg daily intraperitoneally for 28 days	↓mean depth of tumor invasion	not specified	[141]
	Wistar rats injected with C6	8 mg/kg daily orally for different lengths of time, depending on the animals’ lifespan, minimum 13 days, maximum 90 days	↑survival time ↑apoptosis ↓tumor growth	↓MMP-9 protein ↓NF-κB protein	[140]
	SCID mice injected with MNNG/HOS	40, 100 mg/kg five times a week by oral gavage for 24 days	↓tumor volume ↓metastatic incidence in lungs	↓MMP-2 protein	[142]
	NOD-SCID mice injected with HSC-39	10, 20 μM for 3 weeks	↓metastatic incidence in lungs	not specified	[138]
	Balb/c mice injected with H-357	40 mg/kg daily orally for 55 days (resveratrol–nanoparticles)	↓tumor volume	not specified	[137]
	Balb/c nude mice injected with SKBR3/PR	20 mg/kg (free resveratrol) or equivalent dose (resveratrol–solid lipid nanoparticles with or without TPGS), intraperitoneally, 5 times a day for 21 days	↓tumor weight ↓tumor volume	not specified	[136]

Legend: ↑ activation or increase; ↓ inhibition or decreaset.
